# Temporality of clinical factors associated with pancreatic cancer: a case-control study using linked electronic health records

**DOI:** 10.1186/s12885-021-09014-w

**Published:** 2021-11-27

**Authors:** Abu Z. M. Dayem Ullah, Konstantinos Stasinos, Claude Chelala, Hemant M. Kocher

**Affiliations:** 1 Centre for Cancer Biomarkers and Biotherapeutics, Barts Cancer Institute, Queen Mary University of London, London, EC1M 6BQ UK; 2 Barts and the London HPB Centre, The Royal London Hospital, Barts Health NHS Trust, London, UK; 3 Centre for Tumour Biology, Barts Cancer Institute, Queen Mary University of London, London, UK

**Keywords:** Pancreatic cancer, Risk factor, Comorbidity, Lifestyle, Ethnicity

## Abstract

**Background:**

Pancreatic cancer risk is poorly quantified in relation to the temporal presentation of medical comorbidities and lifestyle. This study aimed to examine this aspect, with possible influence of demographics.

**Methods:**

We conducted a retrospective case-control study on the ethnically-diverse population of East London, UK, using linked electronic health records. We evaluated the independent and two-way interaction effects of 19 clinico-demographic factors in patients with pancreatic cancer (*N* = 965), compared with non-malignant pancreatic conditions (*N* = 3963) or hernia (control; *N* = 4355), reported between April 1, 2008 and March 6, 2020. Risks were quantified by odds ratios (ORs) and 95% confidence intervals (CIs) from multivariable logistic regression models.

**Results:**

We observed increased odds of pancreatic cancer incidence associated with recent-onset diabetes occurring within 6 months to 3 years before cancer diagnosis (OR 1.95, 95% CI 1.25-3.03), long-standing diabetes for over 3 years (OR 1.74, 95% CI 1.32-2.29), recent smoking (OR 1.81, 95% CI 1.36-2.4) and drinking (OR 1.76, 95% CI 1.31-2.35), as compared to controls but not non-malignant pancreatic conditions. Pancreatic cancer odds was highest for chronic pancreatic disease patients (recent-onset: OR 4.76, 95% CI 2.19-10.3, long-standing: OR 5.1, 95% CI 2.18-11.9), amplified by comorbidities or harmful lifestyle. Concomitant diagnosis of diabetes, upper gastrointestinal or chronic pancreatic conditions followed by a pancreatic cancer diagnosis within 6 months were common, particularly in South Asians. Long-standing cardiovascular, respiratory and hepatobiliary conditions were associated with lower odds of pancreatic cancer.

**Conclusions:**

Several factors are, independently or via effect modifications, associated with higher incidence of pancreatic cancer, but some established risk factors demonstrate similar magnitude of risk measures of developing non-malignant pancreatic conditions. The findings may inform refined risk-stratification strategies and better surveillance for high-risk individuals, and also provide a means for systematic identification of target population for prospective cohort-based early detection research initiatives.

**Supplementary Information:**

The online version contains supplementary material available at 10.1186/s12885-021-09014-w.

## Background

Pancreatic cancer (PC) was the ninth leading cause of global cancer deaths in 2020 and ranked the 12th most common cancer in the world [1]. The 5-year survival rates of PC patients remain low (3-15%) due to late- or incurable-stage diagnosis [2]. An early-stage diagnosis enables surgical resection of the tumour, and allows increasingly potent adjuvant regimens to improve survival and quality of life [3]. Yet, only 10- 20% of cases are diagnosed at an early-stage [4], while around half are diagnosed at metastatic stage after a meandering presentation with a myriad of non-specific symptoms often leading to an emergency hospital admission [2]. Given that PC-specific symptoms occur late and incidence in general population is low, identifying a suitable high-risk population is preferred over screening of asymptomatic individuals [5, 6].

Although certain demographic, clinical and lifestyle features have been identified as risk factors, such as age, ethnicity, family history, diabetes mellitus, chronic pancreatitis, obesity, dietary factors, tobacco smoking, alcohol abuse, *Helicobacter pylori* infection, and non-O blood group [7, 8], PC aetiology remains elusive even regarding some of the well-established risk factors [9]. It is hypothesised that progression to PC from precancerous non-invasive lesions may take 17 months to 10 years [10], providing a potentially long window for early detection or possible prevention. In particular, well-established risk factors such as diabetes or chronic pancreatitis are first identified in PC patients during the 2 to 3 years window prior to PC diagnosis [6, 9, 11]. The long latency period of PC often follows with rapid deterioration within a space of few months [12], yet studies suggest the tumour remains resectable in asymptomatic patients as late as 6 months before the clinical diagnosis [13, 14]. Taken together, this sequence of tumorigenesis and progression events provide us with a potential for focused screening of high-risk population based on concomitant clinically-relevant features such as comorbidities and lifestyle. In particular, the temporal orientation of comorbidities in relation to PC diagnosis may explain a possible casual to consequential continuum in the reported relationship - from PC initiation by long-standing conditions to reverse causation by PC in the peri-diagnostic period. Considering the complex and multifactorial nature of pancreatic carcinogenesis [15], combination of these clinico-demographic factors can also potentially present with enriched risk measure in some individuals [2], defining a further group suitable for screening.

The majority of the case-control or cohort studies on PC contain predominantly Caucasian (White) populations in Europe or in the USA, limiting their generalisability. In this study, we integrated primary, secondary and tertiary care electronic health records (EHRs) of ~ 5000 patients with pancreatic disease including PC, and ~ 4000 individuals without pancreatic disease from East London, UK. East London is one of the most ethnically and socially diverse local areas in the UK due to its high proportion of immigrant populations, where an estimated 57% of residents belong to a Black, Asian and minority ethnic group [16]. Significant health inequalities exist within the local population including higher rates of diabetes, obesity, liver disease, and other cardiometabolic diseases [17], compared to the wider, more affluent UK population, providing an opportunity for population studies which may have global relevance. We inspected a wide range of medical comorbidities and lifestyle characteristics of the study population, and assessed the potential time-dependent association of these factors with pancreatic cancer development and progression. We also explored all two-way interactions of these factors to identify putative combinations with enriched risk of PC.

## Methods

### Study setting

**Fig. 1:**
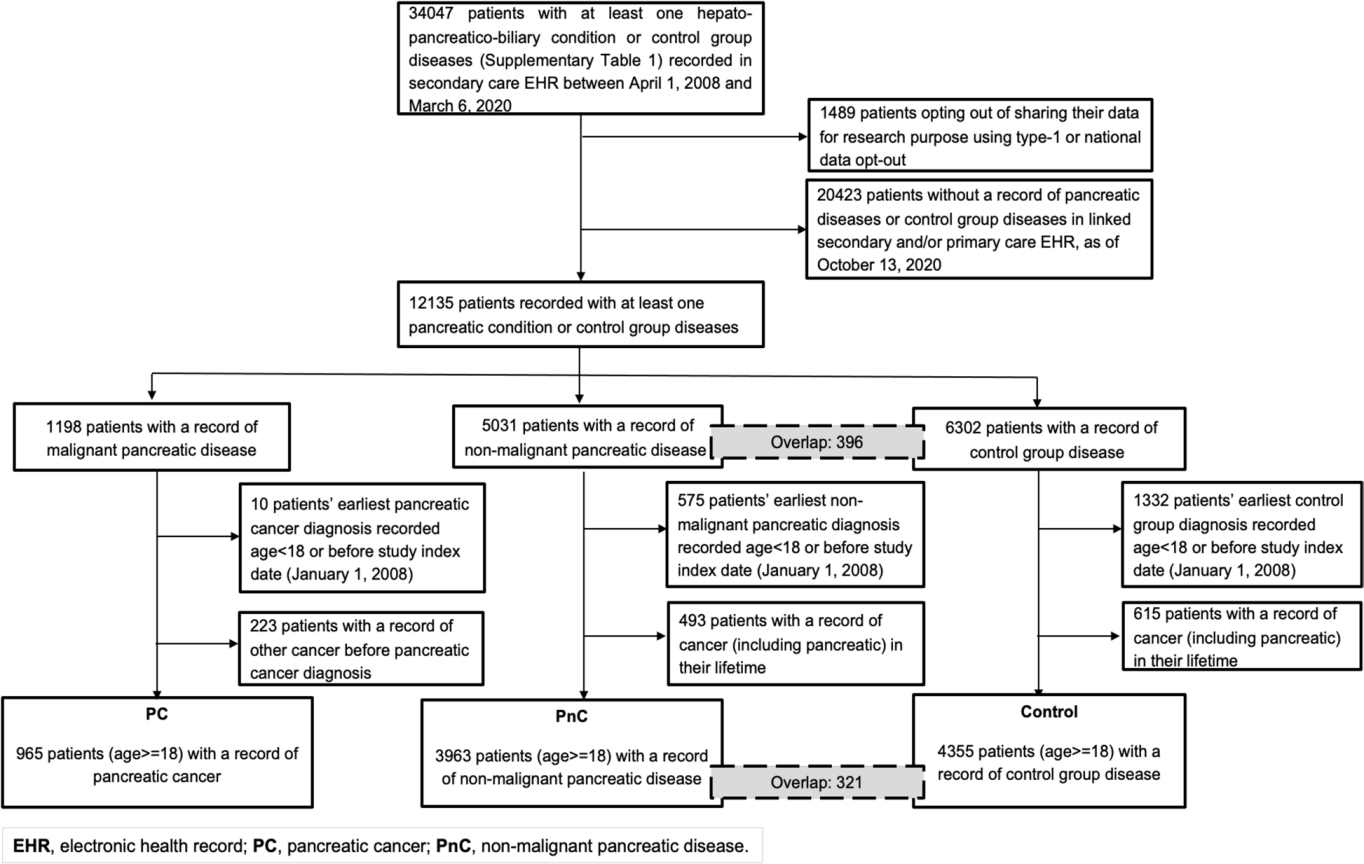
CONSORT diagram for selection of patients in the case-control study

All data utilised for this study were collected and processed under the East London Pancreatic Cancer Epidemiology (EL-PaC-Epidem) study at Barts Health NHS Trust (BHNT). Additional information about the study is provided in supplementary material (Additional file 1 - Methods) and in the study website (https://pac-epidem-el.bcc.qmul.ac.uk). The study collects EHR data on patients diagnosed or reported with hepato-pancreato-biliary (HPB) diseases including cancers as well as patients treated for abdominal hernia at BHNT to be used as “otherwise healthy” contemporaneous comparison cohort (Additional file 1 - Table 1), identified through secondary care EHR and diagnosed between April 1, 2008 and March 6, 2020 (*N* = 34,047; Fig. 1). Care was taken to exclude patients, who have previously requested their GPs or NHS to stop sharing their personal and health records for purposes other than their individual care (*N* = 1489).

### Study design

A case-control study design was adopted to examine the association of PC incidence with demographic features (gender, ethnicity, age); selected comorbidities of interest; and lifestyle factors (smoking, alcohol use, substance misuse, obesity), in comparison to two reference groups: patients with abdominal hernia (control), and non-malignant pancreatic diseases (pancreatic non-cancer; PnC). We explored the association with six common long-term conditions and associated risk factors, with high prevalence in the UK [18, 19]: diabetes, hypertension, hyperlipidaemia, cardiovascular disease, chronic renal disease, and chronic respiratory disease. PC being a disease of gastrointestinal (GI) system with presumably long latent period [12], we also explored its association with non-malignant conditions of upper and lower GI tract as well as pancreas, liver and biliary tract.

The case-control design is particularly suitable to study a disease of low incidence and long latency like PC, and allows investigating its association with multiple putative exposures of interest simultaneously. The choice of two reference groups were driven by the perceived clinical utility of the potential findings. In the hospital settings, PC, PnC and abdominal hernia patients are commonly seen by the same group of clinicians, i.e., HPB or gastroenterology specialist. The baseline characteristic of abdominal hernia allows PC risk assessment with respect to the subgroup of a “general” population, albeit with the challenge of overestimated risk measure [20]. On the contrary, malignant and non-malignant pancreatic diseases demonstrate similar symptomatic presentation [21], therefore identifying any distinctive risk factors may have impact on the evidence-based decision making of the diagnosis/treatment pathway.

### Data collection

The study links secondary or tertiary care EHR from BHNT with primary care EHR through Discovery East London Programme data service. All patient data were harmonised across hospital and GP coding systems where applicable, and organised into 20 variables across four categories (Table 1). The last EHR data extraction date was October 13, 2020. Mortality data was last collected on March 25, 2021.

**Table 1:** Outcomes and variables explored in this study

Group	Variables	Categorisation
Outcome	Incidence	Pancreatic cancer
		Non-malignant pancreatic disease
		Control
Demographic	Gender	Female, Male
	Ethnicity	White, South Asian, Black, Other [, Not known]
	Age group^b^	18-40, 41-50, 51-60, 61-70, 71-80, > 80 years
Comorbidities^a^ *Cardiometabolic and respiratory*	Diabetes	No 0-6 months (peri-diagnosis) > 6-36 months (recent-onset) > 3 years (long-standing)
	Hypertension	
	Hyperlipidaemia	
	Cardiovascular disease	
	Chronic respiratory disease	
	Chronic renal disease	
*Gastrointestinal*	Acute pancreatic disease	
	Chronic pancreatic disease	
	Chronic biliary disease	
	Chronic liver disease	
	Upper GI disease	
	Lower GI disease	
Lifestyle factors^b^	Smoker	Never Past Recent [Not known]
	Alcohol drinker	
	Substance user	
	Obese	

*GI* Gastrointestinal tract

^a^Categorised according to the duration of the disease on the diagnosis date of the outcome category

^b^Categorised according to the representation on the diagnosis date of the outcome category

For each variable, we consulted dictionaries of International Classification of Diseases 10th edition (ICD-10), Systematized Nomenclature of Medicine Clinical Terms (SNOMED CT), Read v2, Clinical Terminology Version 3 (CTV3) or Office of the Population Censuses and Surveys Classification of Interventions and Procedures version 4 (OPCS-4) codes as appropriate to construct the mapping *codelists.* For some variables, *codelists* also included keywords to conduct an automated sub-string search within the semi-structured text as well as local laboratory tests and physiological observation terms. Rule-based phenotyping algorithms were developed for each predictor variable to characterise patients, integrating information from multiple sources where available to counteract bias. A comprehensive list of *codelists* and phenotyping algorithms for the study variables are available on the EL-PaC-Epidem portal (https://pac-epidem-el.bcc.qmul.ac.uk/analysis/).

### Assessment of outcome variable

Participants were divided into incidence outcome groups - PC, PnC, and control, identified by the presence of corresponding ICD-10, SNOMED CT, Read or CTV3 codes assigned in their hospital encounters or GP records during the observation period (April 1, 2008 to October 13, 2020). For each individual within a specific group, the entry date associated with the earliest diagnosis was considered as the date of *index diagnosis* (index date). By design, an individual can belong to both PnC and control groups, and be assigned different index dates depending on the representation; This was done to facilitate examining the association of PC incidence with pre-existing pancreatic conditions in comparison to control group. Patients were excluded from their respective groups if the index diagnosis was made before their 18th birthday or study start date (April 1, 2008). We also excluded individuals from the PC group if they had a record of primary cancer at other sites before the index date. Similarly, we also excluded individuals from the other two groups if they had a record of any cancer in their lifetime, to avoid similar confounding issues. Participants with no record of death were recorded as survivors till the last mortality data collection.

### Assessment of predictor variables

Details on the predictor variables and their categorisation are provided in Additional file 1. Demographic details considered in the study comprised gender (Male, Female); ethnicity (White, South Asian, Black, Other); and age (as 10-year groups: 18-40, 41-50, 51-60, 61-70, 71-80, > 80). Participants were considered to have or have had a specific comorbidity if they met at least one criterion (diagnosis or procedural codes, semi-structured text search, laboratory test results and related medication use) indicating its presence before the index date. Based on the earliest instance of positive record in reference to the index diagnosis, the presence of a medical condition was further temporally stratified into 0-6 months (peri-diagnosis), > 6-36 months (recent-onset) and > 36 months (long-standing) groups. Longitudinal entries about a participant’s lifestyle pattern were derived from diagnosis codes and semi-structured text search. A five-year observation window prior to index date was then used to separate *recent* (i.e., any record of active use/obesity within the window) from *past* status (i.e., active use/obesity prior to that). Patients with no record of a specific lifestyle factor were classified as having missing data.

### Statistical analyses

Descriptive analyses were performed by diagnostic groups: PC, PnC, and control. Differences in demographic characteristics between the groups were assessed using Pearson’s Chi-square test or Kruskal-Wallis rank sum test, as appropriate. To explore the risk factors associated with PC incidence, the effect size for each variable under investigation was evaluated with odds ratios (ORs) with 95% confidence intervals (CI), using multivariable regression models with a binomial distribution. ORs for individual predictor variables (PRD_V) were obtained from independent regression models, controlled for age group, gender, and ethnicity (AGE_V): Model_PRD_V + AGE_V_, and subsequently from a fully-adjusted model controlled for all clinical features: Model_ALL_V_. The modified effect of a predictor variable in interaction with another variable was evaluated in stratified analyses by strata of the second variable from a fully-adjusted regression model. Missing data for ethnicity and lifestyle variables were assigned a separate “Not known” category and included in the respective regression models for effect estimation. In statistical tests, *P* values less than 0.05 were considered significant. All multivariable regression models were separately corrected for multiple testing using Benjamini-Hochberg method and adjusted P values were reported. All statistical analyses and visualisations were performed in R (version 3.5.1). Further details are provided in Additional file 1.

### Sensitivity analyses

We performed several sensitivity analyses to examine the robustness of our results. First, we restricted to individuals for whom both hospital and GP records were available, providing truer ascertainment of the clinical variables. Second, considering the high proportion of missing data for lifestyle variables, we explored the effect of missing data in Model_ALL_V_, in line with previously published methodology [22]. In brief, lifestyle variables were dichotomised into recent and non-recent categories, where participants with past, never and missing status were re-categorised with non-recent status. *Finally*, with a specific interest in distinguishing risk factors of PC from non-malignant pancreatic diseases, we examined the odds of PC compared with the chronic pancreatic condition subgroup consisting of patients with chronic pancreatitis, pancreatic cyst or benign tumour.

## Results

### Baseline characteristics

From the EL-PaC-Epidem cohort, we identified three groups of individuals with pancreatic cancer (PC, *N* = 1198), non-malignant pancreatic diseases (PnC, *N* = 5031), and abdominal hernia (control, *N* = 6302) by the presence of at least one respective diagnosis codes in their hospital encounters or GP records (Fig. 1). After applying exclusion criteria, the final PC risk analysis cohort consisted of 965 patients with PC (81% of the total), 3963 individuals with other pancreatic diseases (79%), and 4355 control subjects (69%) (Fig. 1). Among PC patients, 943 (97.7%) were diagnosed exclusively with exocrine cancer and 15 (1.6%) with endocrine cancer, whereas 7 (0.7%) had both endocrine and exocrine cancer. The non-malignant pancreatic disease group consisted of 2339 (59%) patients diagnosed with chronic pancreatic conditions (CP) including chronic pancreatitis (*N* = 1921), pancreatic cyst (*N* = 629) and benign tumour (*N* = 54); the rest of the patients (*N* = 1624, 41%) had acute pancreatitis without progression to chronic conditions.

Table 2 presents demographic profile of the three participant groups. The PC and PnC groups were male dominated. PC patients were significantly older (median 67.1 years, interquartile range (IQR) 58.9–76.0 years) compared to PnC (median 51.7, IQR 38.6-67.7, *P* < 0.001) and control (median 54.1, IQR 42.0–67.7, *P* < 0.001) patients. All groups had similar majority representation from White communities (between 53.1 and 56.1%), but South Asian representation was notably lower in the PC group (5.6%) than in the PnC group (20.0%). By March 25, 2021, 808 (83.7%) PC patients died with a median survival of 8.1 months (IQR 2.6-18.1 months). The mortality rate in the PnC and control groups during the same period were 20.0 and 14.3% respectively.

**Table 2:** Baseline characteristics of the study groups

	PC (***N*** = 965)	PnC (***N*** = 3963)	Control (***N*** = 4355)	Total^**a**^ (***N*** = 8962)	***P*** value^**b**^ (PC vs PnC)	***P*** value^**b**^ (PC vs Control)
**Gender**					0.851	0.012
Female	453 (46.9%)	1847 (46.6%)	2238 (51.4%)	4373 (48.8%)		
Male	512 (53.1%)	2116 (53.4%)	2117 (48.6%)	4589 (51.2%)		
**Ethnicity**					< 0.001	< 0.001
White	537 (55.6%)	2103 (53.1%)	2442 (56.1%)	4878 (54.4%)		
South Asian	86 (8.9%)	793 (20.0%)	692 (15.9%)	1527 (17.0%)		
Black	100 (10.4%)	366 (9.2%)	671 (15.4%)	1099 (12.3%)		
Other	85 (8.8%)	421 (10.6%)	421 (9.7%)	904 (10.1%)		
Not known	157 (16.3%)	280 (7.1%)	129 (3.0%)	554 (6.2%)		
**Age at diagnosis**^**c**^					< 0.001	< 0.001
Median	67.1	51.7	54.1	55.1		
IQR	58.9, 76.0	38.6, 67.7	42.0, 67.7	41.6, 68.9		
**Diagnosis age group**^**c**^					< 0.001	< 0.001
18-40	26 (2.7%)	1158 (29.2%)	1005 (23.1%)	2149 (24.0%)		
41-50	70 (7.3%)	771 (19.5%)	839 (19.3%)	1611 (18.0%)		
51-60	198 (20.5%)	698 (17.6%)	917 (21.1%)	1736 (19.4%)		
61-70	299 (31.0%)	528 (13.3%)	739 (17.0%)	1522 (17.0%)		
71-80	246 (25.5%)	470 (11.9%)	483 (11.1%)	1142 (12.7%)		
> 80	126 (13.1%)	338 (8.5%)	372 (8.5%)	802 (8.9%)		
**Mortality status**					< 0.001	< 0.001
Deceased	808 (83.7%)	791 (20.0%)	622 (14.3%)	2146 (23.9%)		
Survivor	157 (16.3%)	3172 (80.0%)	3733 (85.7%)	6816 (76.1%)		

^a^There is an overlap of 321 between PnC and Control groups

^b^Differences between groups evaluated by the χ2 test, unless otherwise stated

^c^Differences between groups evaluated by Kruskal-Wallis rank sum test

### Odds of pancreatic cancer (versus control)

**Fig. 2:**
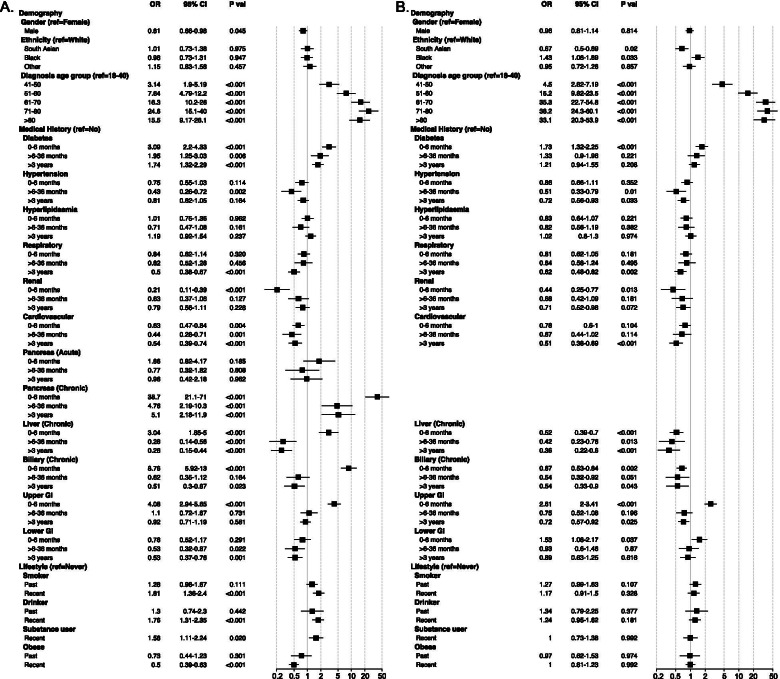
Forest plots showing association between study variables and odds of pancreatic cancer in comparison to: **A** control group; **B** non-malignant pancreatic disease group. The odds ratio (OR) and 95% confidence interval (CI) are derived from fully-adjusted logistic regression model. The reported *P* values are corrected for multiple testing via Benjamini-Hochberg method

We inspected the association between study variables and odds of PC in comparison to the control group (Fig. 2A, Additional file 2 - Table 1). After adjusting for all clinical and demographic variables, we observed a marginally higher odds of PC for women compared to men. PC odds gradually increased with age independent of other clinical characteristics (OR between 3.1 and 24.6), reaching the peak for 71-80 age group following a slight decrease in odds for over 80s. No particular ethnic groups were associated with higher odds of PC.

When compared with controls, PC patients had significantly higher prevalence of diabetes (37.3% vs 24.0%; *P* < 0.001), pancreatic conditions – both acute (5.4% vs 2.6%; *P* < 0.001) and chronic (17.9% vs 1.7%; *P* < 0.001), and chronic biliary diseases (17.8% vs 11.1%; *P* < 0.001), but lower prevalence of chronic respiratory (25.5% vs 34.0%; *P* < 0.001), renal (12.1% vs 15.3%; *P* = 0.013) and lower GI tract (15.5% vs 19.6%; *P* = 0.003) diseases. Expectedly, PC patients had a higher proportion of peri-diagnosis comorbidities reported compared to recent-onset comorbidities, particularly those linked to the digestive system. Consequently, when comorbidities were stratified according to the pre-diagnosis duration, higher PC incidence was strongly associated with peri-diagnosis hepatobiliary, CP and upper GI conditions as well as diabetes.

We also observed higher odds of PC associated with both recent-onset and long-standing diabetes and CP; the inverse association was observed for chronic liver and lower GI conditions. Long-standing cardiovascular and respiratory diseases were also associated with a lower incidence of PC. Recent smoking, drinking and substance misuse were all associated with higher odds of PC; but recent obesity was associated with lower incidence of PC. No significant association was identified for past lifestyle factors.

No significant interaction was observed among demographic characteristics. However, the increase in odds of PC with age (over 50s vs under 40) tended to be higher in White (OR between 12.3 and 31.8), intermediate in Black (OR between 5.0 and 18.4) and moderate in South Asian populations (OR between 6.5 and 8.8); Furthermore, we identified significant odds of PC for the age group 41-50 in White people (OR 4.7, 95% CI 2.1-10.8), but not in Black and South Asian communities (data not shown).

**Fig. 3:**
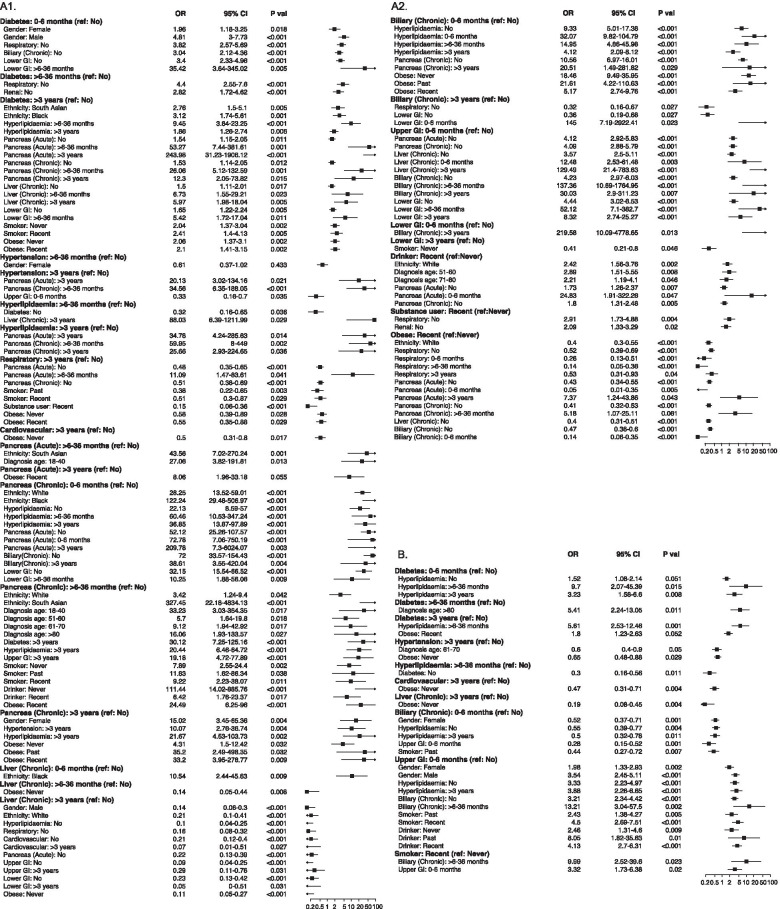
Forest plots showing modified odds of pancreatic cancer associated with study variables among various participant subgroups, in comparison to: **A** control group; **B** non-malignant pancreatic disease group. The odds ratio (OR) and 95% confidence interval (CI) are derived from fully-adjusted logistic regression model. The reported *P* values are corrected for multiple testing via Benjamini-Hochberg method. Only statistically significant effect modifications are shown

There was evidence to suggest that PC risk associated with temporal pattern of several medical comorbidities (compared to the absence of respective conditions) may vary with the status of other clinico-demographic features (Fig. 3A, Additional file 2 - Table 2). Long-standing diabetes was associated with higher odds of PC in patients from South Asian or Black origin. The odds associated with long-standing diabetes also appeared to be amplified in the presence of pancreatic diseases, chronic liver conditions, or hyperlipidaemia. While peri-diagnosis incident diabetes appeared to be a strong indicator of PC, the occurrence was more common in men than women.

The risks for patients with long-standing chronic pancreatic conditions also appeared to be aggravated in women, or in patients with history of hypertension, hyperlipidaemia or obesity. The odds of diagnosing PC following a recent diagnosis of CP was more pronounced in the presence of other comorbidities or harmful lifestyle, or in South Asians. No overall association was observed between acute pancreatitis and PC risk, but recent episode of acute pancreatitis was an indicator in South Asian patients or younger patients below 40.

In general, patients with long-standing diabetes, hypertension or hyperlipidaemia conditions had higher odds of eventually diagnosing with PC when they also experienced acute pancreatic episode in the past or had a recent diagnosis of CP.

### Odds of pancreatic cancer (versus non-malignant pancreatic diseases)

The associations between study variables and odds of PC in comparison with PnC are shown in Fig. 3B. Compared to the White population, the odds of PC was higher in the Black but relatively lower in the South Asian community. The independent higher PC odds associated with increasing age were more pronounced (OR between 4.5 and 38.2). In general, PnC patients had a higher prevalence for majority of the comorbidities (Additional file 2 – Table 3), and accordingly, higher odds of PC diagnosis was associated with peri-diagnosis diabetes, upper GI and lower GI diseases only. Long-standing medical conditions including hepatobiliary, cardiovascular and respiratory problems were more indicative of PnC than PC. No particular lifestyle pattern could clearly differentiate the odds of incidence between PC and PnC.

No significant interaction could be established between ethnicity and other variables in relation to PC, although peri-diagnosis manifestations were more common in South Asian patients (diabetes: OR 3.5, 95% CI 1.5-8.4; upper GI: OR 3.4, 95% CI 1.6-6.9) (data not shown). We observed high-risk interactions among several subgroups which were otherwise independently associated with non-significant or smaller risk of PC compared with PnC (Fig. 3B, Additional file 2 – Table 4). The odds of PC was elevated in patients with long-standing diabetes when they were obese or had developed hyperlipidaemia recently. Recent-onset diabetes appeared to be a significant indicator of PC in patients aged over 80, whereas peri-diagnosis diabetes was more apparent in patients between 50 and 80. Upper GI manifestations of PC were more pronounced in men than women, and in those with the history smoking and drinking. While patients with a recent diagnosis of biliary conditions were more prone to develop PnC, it appeared to be a strong indicator of PC in recent smokers compared to never-smokers.

### Sensitivity analyses

The first sensitivity analyses (SA-1), restricting to patients with both hospital and GP records, did not change the direction of PC odds for all significant covariates with mostly similar estimate. A similar pattern was observed for the second sensitivity analyses (SA-2), in which we dichotomised lifestyle data into *recent* and *non-recent* status and imputed missing data with *non-recent* status. When comparing with the control group (Additional file 3 – Fig. 1), SA-1 yielded an attenuated estimate for age-related PC odds and an elevated odds in patients with diabetes. SA-1 did not reproduce the smaller PC odds for long-standing biliary and recent-onset lower GI conditions. We observed an attenuated estimate of PC odds with recent smoking in SA-2; but the risk associated with recent drinking was attenuated to non-significance. When comparing with PnC (Additional file 3 – Fig. 2), late manifestation with diabetes became more pronounced while recent drinking was associated with marginally higher odds (SA-1). When comparing PC with PnC subgroup with chronic conditions (Additional file 3 – Table 1), the sensitivity analysis reaffirmed the higher odds of PC diagnosis following incident diabetes or upper GI condition, identified new association with recent obesity, but the higher odds associated with patients from Black ethnicity or showing lower GI manifestation attenuated to marginal significance. Unsurprisingly, patients with a history of acute pancreatitis were more likely to develop CP than PC. The overall direction of association for other comorbidities and lifestyle factors remained the same.

## Discussion

We conducted an EHR-led large observational study on an ethnically diverse population and assessed 19 clinico-demographics characteristics simultaneously to identify their effect, or the lack of it, on PC incidence. We have demonstrated the significance of some previously established risk factors, identified the effect of others, and confirmed several two-way combinations as risk factors for PC. In particular, we have assessed the time-dependent association of various medical conditions with PC incidence. While an observational association study such as this cannot imply causality between risk factors and outcome, such temporal stratification is essential to thoroughly interpret and compare findings from different studies. Our results were obtained from statistical models with a conservative method of correcting for multiple comparisons, and robust to various sensitivity analyses except for some variation changing the effect profile from borderline significance to non-significance and vice-versa.

Our study re-affirms the independent effects of diabetes on PC incidence. Consistent with previous studies, for example, the European Study into Digestive Illnesses and Genetics (PanGenEU) and the Pancreatic Cancer Case Control Consortium (PanC4) [9, 23, 24], the odds of PC diagnosis is high within the first few years of incident diabetes, and keeps decreasing but still shows a significant association in the long term. However, our results suggest that recent-onset diabetes alone cannot be considered as any better indicator for manifestation of PC than of pancreatitis or pancreatic cyst. The probability of identifying PC is still higher in the first 6 months after the diagnosis of diabetes, making this a particularly important window from a screening perspective. Similarly, we found high incidence of PC shortly after the diagnosis of CP - reflecting possible misdiagnosis of PC as CP during the initial presentation, and gradual attenuation of the risk measure reflecting the PC-predisposing impact of CP [25].

Somewhat similar to the case with diabetes, we found higher odds of patients diagnosing with PC shortly after identifying diseases of the upper GI tract. However, we could not establish any association with recent-onset gastric conditions and rather demonstrated an increased risk of non-malignant pancreatic disease with long-term gastric conditions. This is in contrast with the majority of observational studies that reported positive associations with a recent history of peptic ulcer [26, 27], gastro-oesophageal reflux disease [26], *H. pylori* infection [26], or long-term diagnosis (up to 20 years) of gastric but not duodenal ulcer [28]. Our findings are not surprising considering the higher prevalence of obese patients in our comparison cohorts, and high body mass index has been shown to be associated with increased risk for various upper GI diseases [29, 30].

The possible synergistic effect of various two-way interactions among clinical factors deserves specific attention. We observed amplified PC odds in patients with recent-onset CP or hyperlipidaemia or obesity on the background of long-standing diabetes. These findings are in line with previous observational studies indicating the possibility of metabolic syndrome driving the risk of PC in a subgroup of patients [26, 31], even though we did not find any positive association with hyperlipidaemia or obesity alone [31]. Similar synergistic effect was observed in CP patients with a history of hyperlipidaemia, smoking, drinking, or obesity issues. Although smoking, alcohol consumption and obesity have all been previously shown as independent risk factors for PC [7, 8], our findings suggest that the risk of developing PC is no greater than the risk of developing other pancreatic conditions first with respect to recent or past lifestyle choice. However, long-term exposure to harmful lifestyle choices appears to increase the sensitivity of the pancreas to be impacted by other diseases augmenting the risk of PC, the examples being smoking with biliary diseases; and obesity with diabetes.

Perhaps, from the screening perspective, any synergistic effect involving patient demographics is more important. In line with previous reports, we also found that the odds of developing PC increases with age, with the highest peak occurring between 60 and 80 years of age [8, 32]. The odds of PC within 6 months of diabetes diagnosis was high for patients over the age of 50, further strengthening the suggestion for a routine check for diabetes in individuals over 50 [6, 33].^.^The case with CP is different, where initial misdiagnosis of PC as CP is common for all age groups, underscoring the need for accurate diagnosis in future screening clinical trials. Our study also evidenced differing PC odds in men and women with long-standing CP, with women being at a five-fold greater odds of PC. Further investigation is required to explain or validate this interaction, as some studies suggest synergistic interactions are more notable for PC development among women [34]. The limited number of PC studies, involving Black, Eastern Asian or Hispanic populations in the USA, suggest that PC risk is considerably higher in Black population than in other ethnic groups [8]. This is reflected in our observed 43% increased risk of PC in the Black compared with the White population. Among the three defined ethnic groups in our study, South Asians appeared to have the lowest risk of PC, but interestingly recent-onset pancreatitis, or concomitant diabetes and upper GI manifestations were more common in this group, suggesting a potential distinctive feature for screening.

Interestingly, long-standing cardiorespiratory or hepatobiliary conditions appeared to be associated with lower odds of PC than other pancreatic diseases. While chronic respiratory conditions such as asthma has been associated with lower risk of PC before [35], further research is warranted to understand if the apparent protective effect can be explained by the involvement of immune or inflammatory factors, and/or by the mediating effect of lifestyle changes following the diagnosis of those health conditions.

A key strength of our study is the use of linked medical records, harmonised for variations in coding that exist between different EHR systems. We ascertained patient demographics, lifestyle, and comorbidities by linking hospital records with longitudinal primary care records, which substantially enrich the data that are recorded on hospital visits. These data have mainly been prospectively registered for administrative and reimbursement purposes, allowing us to obtain a high precision for most of our estimates. Retrospective EHR-based studies often suffer from incomplete or missing data on patient characteristics, particularly for otherwise healthy control patients with low use of healthcare services in the past. Our patient cohort had already been treated or managed at BHNT hospitals at least once, and often referred through primary care, which led to near-complete data for this study, an added advantage of this study. Another strength is the healthy representation of patients from South Asian and Black origin. Considering almost 40% of the world’s population belong to these two ethnic groups, and their growing presence in the Western countries, our results provide a good representation of the global population.

Perhaps, the most notable strength of the current study is the identification of several important combinations of various demographic and clinical features with enriched PC risk estimate. Knowledge of these particular joint effects has practical implications for clinicians, particularly in the primary care settings, to identify patients fitting the profile and refer for advanced screening or specialist consultation in the event of earliest recognised symptoms. The preliminary findings on high-risk interactions also have implications for further PC research, especially in the area of early detection with initiatives based on prospective cohort such as UK-EDI or CPDPC [36, 37], by providing a means for systematic identification of target population and therefore reducing the time and cost of these clinical trials.

Our study has some important limitations. One limitation is the risk of residual confounding due to unmeasured potential confounding variables. For example, the observed association between diabetes and PC risk may be different if the use or non-use of anti-diabetic medication (e.g., metformin, insulin) had been taken into consideration. Similarly, the observed protective effect of several long-standing conditions may change if the disease severity or management strategy were considered. This was partly overcome by controlling for lifestyle changes (e.g., resolving obesity, smoking or alcohol cessation) in the fully-adjusted regression models. A related limitation could be the assessment of obesity as a PC risk factor in comparison with abdominal hernia patients since obesity is considered as a risk factor for abdominal hernia [38]. This could explain the apparent lesser risk of PC for recently obese patients in our study, contrary to the general understanding [7]. Adjustment for deprivation index-based measure of participants’ socio-economic status could further strengthen the demographic dimension of our analysis, although a recent report on the London population suggest that ethnic differences play over and above the level of local area deprivation in explaining education, employment, housing and health outcomes [39].

Another critical limitation is associated with the absence of primary care data for a proportion of the study participants. The study currently has ethical approval to collect primary care data from East London. However, the Royal London Hospital, one of the five hospitals under BHNT, hosts one of the largest HPB centres in England, and provides specialist support for patients with suspected or confirmed severe HPB conditions from East London as well as nearby geographical areas. Hence, primary care data was missing in approximately 41, 23 and 13% of PC, PnC and control groups respectively. The final analysis cohort also had high proportion of missing data for lifestyle variables - approximately 20, 41 and 21% missing data for smoking, drinking and obesity variables respectively, even though these were considerably lower than those reported in a primary care EHR database of the representative UK population (35, 47, and 48% respectively) [40]. However, our sensitivity analyses suggest that this is unlikely to substantially impact our findings, mainly due to the detailed semi-structured text entries (discharge summaries, past medical history and a lifestyle questionnaire collected during the pre-operative assessment, and presenting symptoms from scheduled or unscheduled hospital visits) from BHNT that were incorporated in the study.

## Conclusions

Based on an EHR-led large observational study, we showed that several clinical factors are, independently or via effect modifications, associated with higher incidence of pancreatic cancer, whereas some established risk factors have similar risk measures of developing non-malignant pancreatic conditions. The findings are evidence in support of developing PC risk-stratification strategies from routine medical records through the assessment of individuals’ demographic, comorbidity and lifestyle profile. When it comes to implementation in the clinical settings, the identified risk factors can be used as indication for primary care-based surveillance, if not serving as the first sieve in a multi-stage, targeted screening model [41], with a realistic expectation of identifying patients with “silent” non-cancerous pancreatic conditions in the process. Considering the well-documented probability of progression to PC from pancreatitis and cystic neoplasm [2, 3, 42, 43], early detection of these patient groups and taking measures such as extirpation of cystic neoplasms or limitation of smoking and alcohol use [8], can be considered as a PC preventive strategy serving a broader goal.

This study is one of the several arms under the umbrella EL-PaC-Epidem project, the scope of which extends to additional longitudinal information about participants such as reported symptoms, prescriptions drugs usage, pathology test results, and healthcare utilisation markers. The end goal is to develop a machine learning based prediction tool with high discriminatory ability of identifying a high-risk group of individuals for PC screening and surveillance programs from routine EHR, leading to increased early diagnosis and contributing to improved survival in this otherwise dismal disease. This study is an important first step towards that goal.

## Supplementary Information

**Additional file 1: **Supplementary Methods. **Table 1.** Diagnosis groups of participants for East London Pancreatic Cancer Epidemiology study.

**Additional file 2: **Supporting Results. **Table 1.** Association between study variables and odds of pancreatic cancer in comparison to control group. **Table 2.** Modified odds of pancreatic cancer associated with individual medical condition and lifestyle factors among different participant subgroups, in comparison to control group. **Table 3.** Association between study variables and odds of pancreatic cancer in comparison to non-malignant pancreatic disease group. **Table 4.** Modified odds of pancreatic cancer associated with individual medical condition and lifestyle factors among different participant subgroups, in comparison to non-malignant pancreatic disease group.

**Additional file 3: **Sensitivity Analyses Results. **Fig 1.** Sensitivity analysis: Forest plots showing association between study variables and odds of pancreatic cancer in comparison to control group. **Fig 2.** Sensitivity analysis: Forest plots showing association between study variables and odds of pancreatic cancer in comparison to non-malignant pancreatic disease group. **Table 1.** Sensitivity analysis: Association between study variables and odds of pancreatic cancer in comparison to chronic pancreatic conditions group.

## Data Availability

All statistical data relevant to the study are included in the article or uploaded as supplementary information. Only the corresponding author has full access to all the participants’ data in the study. The authors confirm that the completely anonymised final analysis dataset for this work are available from the corresponding author upon reasonable request.

## Abbreviations

CI: Confidence interval
CP: Chronic pancreatic diseases
EHR: Electronic health record
GI: Gastrointestinal
HPB: Hepato-pancreato-biliary
OR: Odds ratio
PC: Pancreatic cancer
PnC: Non-malignant diseases of pancreas
