# Supplementary material for: Temporality of clinical factors associated with pancreatic cancer: a case-control study using linked electronic health records
Source: BMC Cancer. 2021 Nov 27;21:1279. doi: 10.1186/s12885-021-09014-w (PMC8626898; doi:10.1186/s12885-021-09014-w)
Supplement: Supplementary file 1 — Additional file 1: Supplementary Methods. Table 1. Diagnosis groups of participants for East London Pancreatic Cancer Epidemiology study. [file 12885_2021_9014_MOESM1_ESM.pdf]

Contents

Methods 2

Table 1 Diagnosis groups of participants for East London Pancreatic Cancer Epidemiology study. 10

References 11

METHODS

Study setting

All data utilised for this study were collected and processed under the East London Pancreatic Cancer Epidemiology (EL-PaC-Epidem) study at Barts Health NHS Trust (BHNT). BHNT is the largest National Health Service (NHS) Trust in England and acts as a provider of secondary care facilities for around 2.5 million population of East London (circa 50% from 97 nationalities) as well as a range of tertiary care services [1]. In brief, EL-PaC-Epidem is an ongoing study that ascertains patients diagnosed or reported with hepato-pancreato-biliary (HPB) diseases including cancers as well as patients treated for abdominal hernia to be used as “otherwise healthy” contemporaneous comparison cohort (Table 1), within five BHNT hospital sites (The Royal London Hospital, Newham University Hospital, St Bartholomew's Hospital, Whipps Cross University Hospital, Mile End Hospital) between 2008 and 2022. Patients, who have previously informed their General Practitioners (GPs) or National Health Services (NHS) UK to stop sharing their personal and health records for purposes other than their individual care, are automatically excluded from any subsequent data collection and analysis processes. The study was approved by the East of England - Essex Research Ethics Committee (19/EE/0163; May 17, 2019) and supported by the NHS Confidentiality Advisory Group for collecting and processing confidential patient information without consent (19/CAG/0219; January 17, 2020). The study protocol was approved by the Queen Mary University of London and Barts Health NHS Trust Joint Research Management Office on February 5, 2019 (Ref 249837; https://pac-epidem-el.bcc.qmul.ac.uk/methods.html). The study is limited to the secondary use of a specified subset of patients’ retrospective electronic health

record (EHR) generated during the course of normal care of these patients. It links EHRs from different data sources (via UK unique individual NHS numbers), including primary care through GP (Discovery East London Programme data service [DDS]) and secondary or tertiary care through hospitals (BHNT Consolidated Data Extract [CDE]).

Data collection

All patient data were obtained from retrospective EHR, harmonised across hospital and GP coding systems where applicable, and organised into 20 primary variables across four categories corresponding to the focus of the study. BHNT CDE uses 2011 UK census grouping to record ethnicity, International Classification of Diseases 10th edition (ICD-10) or Systematized Nomenclature of Medicine Clinical Terms (SNOMED CT) diagnosis codes for clinically relevant diagnoses, and Office of the Population Censuses and Surveys Classification of Interventions and Procedures version 4 (OPCS-4) procedural codes for treatments and procedures. Physiological observations (weight, body-mass index [BMI], blood pressure) and laboratory tests results are available in locally developed terms. Semi-structured text entries such as discharge summaries, past medical history and a lifestyle questionnaire collected during the pre-operative assessment, and presenting symptoms from scheduled or unscheduled hospital visits are also available. All GP records via DDS were available in Read Codes v2 or Clinical Terminology Version 3 (CTV3) codes, except the prescribed medication records which were available in SNOMED codes. For each variable, we consulted ICD-10, SNOMED, Read, CTV3 or OPCS-4 dictionaries as appropriate to construct the mapping *codelists.* For some variables, codelists also included keywords to conduct automated sub-string search within semi-structured text as well as local laboratory test and physiological observation terms. Rule-based

phenotyping algorithms were developed for each predictor variable to characterise patients, integrating information from multiple sources where available to counteract bias. A comprehensive list of *codelists* and phenotyping algorithms for the study variables are available on the EL-PaC-Epidem portal (https://pac-epidem-el.bcc.qmul.ac.uk/analysis/).

Assessment of outcome variable

Participants were divided into three incidence outcome groups – Pancreatic cancer (PC), non-malignant pancreatic diseases (pancreatic non-cancer; PnC), and hernia (control) - identified by the presence of corresponding ICD-10/SNOMED CT diagnoses codes assigned in their hospital encounters, or Read/CTV3 codes assigned in their GP records during the observation period (April 1, 2008 to October 13, 2020). For each individual within a specific group, the entry date associated with the earliest diagnosis was considered as *index date*, i.e., the date of index diagnosis. By design, an individual can belong to both PnC and control groups, and be assigned different index dates depending on the representation; This was done to facilitate examining the association of PC incidence with pre-existing pancreatic conditions in comparison to control group. Patients were excluded from their respective groups if the index diagnosis was made before their 18th birthday or study start date (April 1, 2008). Individuals were excluded from the PC group if they had a record of primary cancer at other sites before the index date. Similarly, individuals were excluded from the other two groups if they had a record of any cancer in their lifetime, to avoid similar confounding issues.

Assessment of predictor variables

Rule-based phenotyping algorithms were developed for each predictor variable to characterise patients, integrating information from multiple sources. Ethnicity was grouped into four categories - White, South Asian, Black, and Other. White and Black ethnic groups were defined based on the 2011 UK census classification; Indian, Pakistani and Bangladeshi origin from the Asian group represented South Asian, while the rest (i.e., Mixed, Chinese, other Asian and other ethnic group) were represented in the Other group. The ethnic category recorded at the GP took precedence over hospital records.

Phenotyping algorithms defining the comorbidities were based on diagnosis codes (presence) or semi-structured text search (presence or absence), with the additional inclusion of procedural codes (presence), some observation or laboratory test results (presence) and related medication use (at least three prescriptions). Patients were considered to have or have had a specific medical condition if they met at least one criterion indicating the presence of the condition before the *index date* (earliest diagnosis date to represent an outcome incidence group), otherwise they were considered negative for the condition. Based on the earliest instance of positive record in reference to the index date, the presence of a medical condition was further temporally stratified into 0-6 months (peri-diagnosis), >6-36 months (recent-onset) and >36 months (long-standing) groups. Thus, a participant who had the earliest record of diabetes within the six-month period before PC diagnosis was assigned with peri-diagnosis diabetes, whereas a participant from PnC group was assigned with long-standing hypertension if hypertension was evidenced at least three years before the non-malignant pancreatic condition diagnosis.

Phenotyping algorithms defining the lifestyle factors were based on the longitudinal entries (current, past or never) derived from diagnosis codes and semi-structured text search, with the additional inclusion of BMI observation for obesity. Obesity was defined as BMI of 30 kg/m^2 or more. Patients assigned never status at any point but having a record of current or past status before that date were reassigned to past status. In order to accommodate any remaining deleterious effects of an unhealthy lifestyle choice after quitting/resolving, a five-year observation window prior to index date was then used to group patients with *recent* and *past* categories. Patients with any record of current status within the observation window were assigned to *recent* category. Patients were assigned to *past* category, if they were not already assigned to *recent* category, and i) the observation window contained entries with past status, or ii) the window contained no entries and the latest status prior to the window was current or past. Patients with never status only were assigned to *never* category. Patients with no record of a specific lifestyle factor were classified as having missing data.

Statistical analyses

Descriptive analyses were performed by diagnostic groups: PC, PnC, and control. There were 965 PC cases, 3963 patients with PnC conditions and 4355 controls available for assessing the risk factors associated with PC incidence. There was an overlap of 321 between PnC and controls. Differences in demographic characteristics between the groups by case-reference status, i.e., PC-PnC and PC-control, were assessed using Pearson's Chi-square test or Kruskal-Wallis rank sum test, as appropriate.

To explore the risk factors associated with PC incidence, the effect size for each variable under investigation was evaluated with odds ratios (ORs) with 95% confidence intervals (CI), using multivariable regression models. ORs for individual demographic, comorbidity and lifestyle predictor variables (PRD_V) were obtained from independent regression models, controlled for demographic variables, i.e., age group, gender, and ethnicity ($Model_{PRD\_V+AGE\_V}$), and subsequently from a single fully-adjusted model simultaneously controlled for all predictor variables ($Model_{ALL\_V}$). As such, $Model_{PRD\_V+AGE\_V}$ to obtain ORs for gender, ethnicity, and age group variables essentially represent a single model $Model_{AGE\_V}$.

The modified effect associated with interaction between any two predictor variables was obtained from stratified analysis, where effect size for the first variable was evaluated by strata of the second variable (i.e., stratification variable; STR_V): for a target predictor variable with *n* categories, the ORs for each *n*-1 non-reference category are calculated from separate fully-adjusted models, with target variable converted into a binary treatment variable (TRT_V: 0 = reference category, 1 = non-reference category) and stratification variable excluded ($Model_{ALL\_V\text{-}STR\_V}$). The P value for interaction between the stratification variable and the binary treatment variable was obtained with a likelihood ratio test comparing the main regression analysis model ($Model_{ALL\_V}$) with a model that has an additional interaction term ($Model_{ALL\_V+\ TRT\_V:STR\_V}$).

Missing data for ethnicity and lifestyle variables were assigned a separate “Not known” category and included in the respective regression models for effect estimation. In all statistical tests, P values less than 0.05 were considered significant. All multivariable regression models were separately corrected for multiple testing using Benjamini-

Hochberg method and adjusted P values were reported. All statistical analyses and visualisations were performed in R (version 3.5.1).

Sensitivity analyses

We performed several sensitivity analyses to examine the robustness of our results. *First*, we restricted to individuals for whom both hospital and GP records were available, providing truer ascertainment of the clinical variables. This reduced our analysis cohort to 566 PC, 3038 PnC and 3793 control patients, a reduction of approximately 41%, 23% and 13% respectively. The fully-adjusted regression model $\text{Model}_{\text{ALL\_V}}$ was used to obtain ORs associated with the predictor variables. *Second*, considering the high proportion of missing data for lifestyle variables, we explored the effect of missing data. The final analysis cohort had high proportion of missing data for lifestyle variables - approximately 20%, 41% and 21% missing data for smoking, drinking and obesity variables respectively, even though these were considerably lower than those reported in a primary care EHR database of the representative UK population (35%, 47%, and 48% respectively) [2]. An earlier study on the quality of recorded smoking status suggests that current smokers may be adequately identified using EHR, whereas those with no smoking status are more likely to be past smokers or never smokers than current smokers [3]. In line with their reasoning, we explored the effect of missing lifestyle data in $\text{Model}_{\text{ALL\_V}}$ by adopting a simplified dichotomous presentation of lifestyle variables with *recent* and *non-recent* categories, where participants with past, never and missing categories were collapsed into *non-recent* category. *Finally*, with a specific interest in distinguishing risk factors of PC from non-malignant pancreatic diseases, we examined the odds of PC compared with the

chronic pancreatic condition subgroup (n=2339) consisting of patients with chronic pancreatitis, or pancreatic cyst or benign tumour.

Table 1 Diagnosis groups of participants for East London Pancreatic Cancer Epidemiology study.

Group	Diagnosis
Pancreatic cancer	Malignant neoplasm of pancreas<br>Carcinoma in situ of pancreas<br>Neoplasm of uncertain behaviour of pancreas
Non-malignant pancreatic disease	Benign neoplasm of pancreas<br>Acute pancreatitis<br>Other diseases of pancreas *including* chronic pancreatitis and pancreatic cyst
Hepatobiliary cancer	Malignant neoplasm of liver and intrahepatic bile ducts<br>Malignant neoplasm of gallbladder<br>Malignant neoplasm of other and unspecified parts of biliary tract<br>Carcinoma in situ of liver, gallbladder and bile ducts<br>Neoplasm of uncertain or unknown behaviour of liver, gallbladder and bile ducts
Non-malignant hepatobiliary disease	Benign neoplasm of Liver<br>Benign neoplasm of Extrahepatic bile ducts<br>Alcoholic liver diseases<br>Toxic liver disease<br>Hepatic failure<br>Chronic hepatitis<br>Fibrosis and cirrhosis of liver<br>Other inflammatory liver diseases<br>Other diseases of liver *including* fatty liver<br>Cholelithiasis<br>Cholecystitis<br>Other diseases of gallbladder<br>Other diseases of biliary tract *including* cholangitis and biliary cyst
Control	Umbilical hernia<br>Ventral hernia<br>Other or unspecified abdominal hernia[†]

† Inguinal hernia, being predominantly observed in male, is not included

References

[1] Barts Health NHS Trust. https://www.bartshealth.nhs.uk/about-us (accessed August 10, 2020)

[2] Nicholson BD, Aveyard P, Bankhead CR, Hamilton W, Hobbs FDR, and Lay-Flurrie S. Determinants and extent of weight recording in UK primary care: an analysis of 5 million adults' electronic health records from 2000 to 2017. *BMC Med.* 2019; **17**(1):222.

[3] Marston L, Carpenter JR, Walters KR, Morris RW, Nazareth I, White IR, et al. Smoker, ex-smoker or non-smoker? The validity of routinely recorded smoking status in UK primary care: a cross-sectional study. *BMJ Open* 2014; **4**(4):e004958.
