# Supplementary material for: Temporality of clinical factors associated with pancreatic cancer: a case-control study using linked electronic health records
Source: BMC Cancer. 2021 Nov 27;21:1279. doi: 10.1186/s12885-021-09014-w (PMC8626898; doi:10.1186/s12885-021-09014-w)
Supplement: Supplementary file 2 — Additional file 2: Supporting Results. Table 1. Association between study variables and odds of pancreatic cancer in comparison to control group. Table 2. Modified odds of pancreatic cancer associated with individual medical condition and lifestyle factors among different participant subgroups, in comparison to control group. Table 3. Association between study variables and odds of pancreatic cancer in comparison to non-malignant pancreatic disease group. Table 4. Modified odds of pancreatic cancer associated with individual medical condition and lifestyle factors among different participant subgroups, in comparison to non-malignant pancreatic disease group. [file 12885_2021_9014_MOESM2_ESM.pdf]

Contents

Table 1 Association between study variables and odds of pancreatic cancer in comparison to control group. 2

Table 2 Modified odds of pancreatic cancer associated with individual medical condition and lifestyle factors among different participant subgroups, in comparison to control group. 5

Table 3 Association between study variables and odds of pancreatic cancer in comparison to non-malignant pancreatic disease group. 17

Table 4 Modified odds of pancreatic cancer associated with individual medical condition and lifestyle factors among different participant subgroups, in comparison to non-malignant pancreatic disease group. 20

Table 1 Association between study variables and odds of pancreatic cancer in comparison to control group.

Demographic	PC (N=965)	Control (N=4355)	$Model_{PRD\_V+AGE\_V}$ OR (95% CI)	$Model_{PRD\_V+AGE\_V}$ P value	$Model_{ALL\_V}$ OR (95% CI)	$Model_{ALL\_V}$ P value
**Gender**						
Female	453 (46.9%)	2238 (51.4%)	Ref.		Ref.	
Male	512 (53.1%)	2117 (48.6%)	1.04 (0.9-1.22)	0.701	0.81 (0.66-0.98)	0.049
**Ethnicity**						
White	537 (55.6%)	2442 (56.1%)	Ref.		Ref.	
South Asian	86 (8.9%)	692 (15.9%)	0.69 (0.54-0.89)	0.009	1.01 (0.73-1.38)	0.982
Black	100 (10.4%)	671 (15.4%)	0.85 (0.67-1.08)	0.318	0.98 (0.73-1.31)	0.965
Other	85 (8.8%)	421 (9.7%)	1.18 (0.9-1.53)	0.318	1.15 (0.83-1.58)	0.467
Not known	157 (16.3%)	129 (3.0%)	5.57 (4.25-7.33)	<0.001	4.11 (2.92-5.8)	<0.001
**Diagnosis age group**						
18-40	26 (2.7%)	1005 (23.1%)	Ref.		Ref.	
41-50	70 (7.3%)	839 (19.3%)	3.17 (2.02-5.12)	<0.001	3.14 (1.9-5.19)	<0.001
51-60	198 (20.5%)	917 (21.1%)	8.05 (5.37-12.6)	<0.001	7.64 (4.79-12.2)	<0.001
61-70	299 (31.0%)	739 (17.0%)	15 (10.1-23.3)	<0.001	16.3 (10.2-26)	<0.001
71-80	246 (25.5%)	483 (11.1%)	18.1 (12.1-28.3)	<0.001	24.6 (15.1-40)	<0.001
>80	126 (13.1%)	372 (8.5%)	12.8 (8.36-20.4)	<0.001	15.5 (9.17-26.1)	<0.001
**Comorbidities**						
**Diabetes**						
No	605 (62.7%)	3310 (76.0%)	Ref.		Ref.	
0-6 months	140 (14.5%)	154 (3.5%)	3.24 (2.47-4.24)	<0.001	3.09 (2.2-4.33)	<0.001
>6-36 months	44 (4.6%)	165 (3.8%)	1.4 (0.98-2.01)	0.137	1.95 (1.25-3.03)	0.006
>3 years	176 (18.2%)	726 (16.7%)	1.04 (0.85-1.28)	0.809	1.74 (1.32-2.29)	<0.001
**Hypertension**						
No	396 (41.0%)	1849 (42.5%)	Ref.		Ref.	
0-6 months	150 (15.5%)	323 (7.4%)	0.97 (0.75-1.26)	0.9	0.75 (0.55-1.03)	0.12
>6-36 months	32 (3.3%)	309 (7.1%)	0.34 (0.23-0.51)	<0.001	0.43 (0.26-0.72)	0.002
>3 years	387 (40.1%)	1874 (43.0%)	0.51 (0.42-0.61)	<0.001	0.81 (0.62-1.05)	0.172
**Hyperlipidaemia**						
No	524 (54.3%)	2507 (57.6%)	Ref.		Ref.	
0-6 months	148 (15.3%)	463 (10.6%)	0.92 (0.74-1.16)	0.619	1.01 (0.75-1.36)	0.979
>6-36 months	50 (5.2%)	292 (6.7%)	0.61 (0.44-0.85)	0.008	0.71 (0.47-1.08)	0.169
>3 years	243 (25.2%)	1093 (25.1%)	0.72 (0.6-0.87)	0.002	1.19 (0.92-1.54)	0.248
**Respiratory**						
No	719 (74.5%)	2876 (66.0%)	Ref.		Ref.	
0-6 months	106 (11.0%)	389 (8.9%)	0.8 (0.62-1.02)	0.135	0.84 (0.62-1.14)	0.334
>6-36 months	42 (4.4%)	220 (5.1%)	0.68 (0.48-0.97)	0.068	0.82 (0.52-1.28)	0.465
>3 years	98 (10.2%)	870 (20.0%)	0.41 (0.33-0.52)	<0.001	0.5 (0.38-0.67)	<0.001
**Renal**						

No	848 (87.9%)	3690 (84.7%)	Ref.		Ref.	
0-6 months	18 (1.9%)	141 (3.2%)	0.33 (0.19-0.55)	<0.001	0.21 (0.11-0.39)	<0.001
>6-36 months	26 (2.7%)	150 (3.4%)	0.56 (0.36-0.87)	0.017	0.63 (0.37-1.06)	0.134
>3 years	73 (7.6%)	374 (8.6%)	0.64 (0.48-0.84)	0.003	0.79 (0.56-1.11)	0.239
**Cardiovascular**						
No	714 (74.0%)	3211 (73.7%)	Ref.		Ref.	
0-6 months	127 (13.2%)	411 (9.4%)	0.71 (0.56-0.9)	0.01	0.63 (0.47-0.84)	0.004
>6-36 months	37 (3.8%)	230 (5.3%)	0.42 (0.29-0.61)	<0.001	0.44 (0.28-0.71)	0.002
>3 years	87 (9.0%)	503 (11.5%)	0.43 (0.33-0.56)	<0.001	0.54 (0.39-0.74)	<0.001
**Pancreas (Acute)**						
No	913 (94.6%)	4241 (97.4%)	Ref.		Ref.	
0-6 months	27 (2.8%)	21 (0.5%)	6.98 (3.79-12.9)	<0.001	1.86 (0.82-4.17)	0.194
>6-36 months	12 (1.2%)	42 (1.0%)	1.81 (0.92-3.57)	0.174	0.77 (0.32-1.82)	0.619
>3 years	13 (1.3%)	51 (1.2%)	1.51 (0.78-2.9)	0.281	0.96 (0.42-2.18)	0.979
**Pancreas (Chronic)**						
No	792 (82.1%)	4282 (98.3%)	Ref.		Ref.	
0-6 months	143 (14.8%)	17 (0.4%)	42.8 (25-73.5)	<0.001	38.7 (21.1-71)	<0.001
>6-36 months	17 (1.8%)	34 (0.8%)	2.89 (1.54-5.42)	0.002	4.76 (2.19-10.3)	<0.001
>3 years	13 (1.3%)	22 (0.5%)	3.96 (1.89-8.28)	<0.001	5.1 (2.18-11.9)	<0.001
**Liver (Chronic)**						
No	862 (89.3%)	3864 (88.7%)	Ref.		Ref.	
0-6 months	70 (7.3%)	57 (1.3%)	4.7 (3.19-6.95)	<0.001	3.04 (1.85-5)	<0.001
>6-36 months	14 (1.5%)	187 (4.3%)	0.37 (0.21-0.64)	<0.001	0.28 (0.14-0.56)	<0.001
>3 years	19 (2.0%)	247 (5.7%)	0.32 (0.2-0.52)	<0.001	0.26 (0.15-0.44)	<0.001
**Biliary (Chronic)**						
No	793 (82.2%)	3870 (88.9%)	Ref.		Ref.	
0-6 months	134 (13.9%)	67 (1.5%)	8.52 (6.13-11.8)	<0.001	8.76 (5.92-13)	<0.001
>6-36 months	18 (1.9%)	195 (4.5%)	0.48 (0.29-0.8)	0.009	0.62 (0.35-1.12)	0.173
>3 years	20 (2.1%)	223 (5.1%)	0.44 (0.28-0.71)	0.002	0.51 (0.3-0.87)	0.025
**Upper GI**						
No	644 (66.7%)	3005 (69.0%)	Ref.		Ref.	
0-6 months	144 (14.9%)	172 (3.9%)	3.49 (2.69-4.53)	<0.001	4.08 (2.94-5.65)	<0.001
>6-36 months	43 (4.5%)	261 (6.0%)	0.79 (0.56-1.12)	0.287	1.1 (0.72-1.67)	0.744
>3 years	134 (13.9%)	917 (21.1%)	0.65 (0.53-0.8)	<0.001	0.92 (0.71-1.19)	0.592
**Lower GI**						
No	815 (84.5%)	3500 (80.4%)	Ref.		Ref.	
0-6 months	66 (6.8%)	178 (4.1%)	1.28 (0.94-1.75)	0.184	0.78 (0.52-1.17)	0.304
>6-36 months	29 (3.0%)	214 (4.9%)	0.5 (0.33-0.76)	0.003	0.53 (0.32-0.87)	0.024
>3 years	55 (5.7%)	463 (10.6%)	0.48 (0.36-0.65)	<0.001	0.53 (0.37-0.76)	0.001
**Lifestyle**						
**Smoker**						
Never	257 (26.6%)	1889 (43.4%)	Ref.		Ref.	
Past	194 (20.1%)	941 (21.6%)	1.08 (0.87-1.35)	0.665	1.28 (0.98-1.67)	0.118

Recent	188 (19.5%)	944 (21.7%)	1.74 (1.38-2.18)	<0.001	1.81 (1.36-2.4)	<0.001
Not known	326 (33.8%)	581 (13.3%)	2.9 (2.34-3.59)	<0.001	2.76 (2.04-3.74)	<0.001
**Drinker**						
Never	135 (14.0%)	1195 (27.4%)	Ref.		Ref.	
Past	27 (2.8%)	161 (3.7%)	1.35 (0.85-2.15)	0.312	1.3 (0.74-2.3)	0.451
Recent	289 (29.9%)	1294 (29.7%)	1.87 (1.47-2.39)	<0.001	1.76 (1.31-2.35)	<0.001
Not known	514 (53.3%)	1705 (39.2%)	2.19 (1.75-2.74)	<0.001	1.49 (1.13-1.98)	0.011
**Substance user**						
Never	132 (13.7%)	1428 (32.8%)	Ref.		Ref.	
Past	0 (0.0%)	8 (0.2%)	NA	0.98	NA	0.982
Recent	130 (13.5%)	533 (12.2%)	1.62 (1.22-2.16)	0.002	1.58 (1.11-2.24)	0.022
Not known	703 (72.8%)	2386 (54.8%)	2.66 (2.15-3.28)	<0.001	2.69 (2.06-3.49)	<0.001
**Obese**						
Never	452 (46.8%)	1781 (40.9%)	Ref.		Ref.	
Past	30 (3.1%)	119 (2.7%)	0.72 (0.47-1.1)	0.26	0.73 (0.44-1.23)	0.314
Recent	214 (22.2%)	1616 (37.1%)	0.46 (0.38-0.56)	<0.001	0.5 (0.39-0.63)	<0.001
Not known	269 (27.9%)	839 (19.3%)	0.9 (0.74-1.09)	0.409	0.58 (0.45-0.76)	<0.001

The reported P values are corrected for multiple testing via Benjamini-Hochberg method.
$Model_{PRD\_V+AGE\_V}$: Adjusted for gender, ethnicity, and diagnosis age group variables (AGE_V) for each individual predictor variable (PRD_V)
$Model_{ALL\_V}$: Simultaneously adjusted for all demographic, comorbidities and lifestyle factors
PC, pancreatic cancer; GI, gastrointestinal tract; OR, odds ratio; CI, confidence interval; NA, not available

Table 2 Modified odds of pancreatic cancer associated with individual medical condition and lifestyle factors among different participant subgroups, in comparison to control group.

		Diabetes (Ref. No)								
		0-6 months			>6-36 months			>3 years		
**Modifier**	**Subgroup**	**OR (95% CI)**	**P value**	**P-inter**	**OR (95% CI)**	**P value**	**P-inter**	**OR (95% CI)**	**P value**	**P-inter**
Gender	Female	1.96 (1.18-3.25)	0.018	0.009	2.59 (1.35-4.97)	0.025	0.656	1.46 (1-2.12)	0.076	0.202
	Male	4.81 (3-7.73)	<0.001		2.12 (1.14-3.95)	0.056		1.97 (1.38-2.81)	0.001	
Ethnicity	White	3.47 (2.21-5.45)	<0.001	0.244	2.24 (1.23-4.1)	0.036	0.733	1.36 (0.94-1.97)	0.148	0.002
	South Asian	6.06 (2.13-17.23)	0.002		2.52 (0.7-9.06)	0.24		2.76 (1.5-5.1)	0.005	
	Black	4.23 (1.57-11.42)	0.009		2.65 (0.9-7.77)	0.14		3.12 (1.74-5.61)	0.001	
Diagnosis age group	18-40			0.417			0.093	5.71 (1.5-21.83)	0.023	0.172
	41-50	2.49 (0.54-11.49)	0.299		3.77 (1.1-12.91)	0.087		2.79 (1.1-7.12)	0.055	
	51-60	4.35 (2.23-8.47)	<0.001		2.35 (1.01-5.47)	0.112		2.17 (1.27-3.7)	0.012	
	61-70	4.29 (2.32-7.94)	<0.001		1.53 (0.65-3.61)	0.44		1.24 (0.77-1.98)	0.456	
	71-80	2.32 (1.12-4.8)	0.041		0.53 (0.1-2.68)	0.544		1.41 (0.87-2.27)	0.223	
	>80	1.88 (0.8-4.43)	0.203		5.79 (2.27-14.73)	0.003		2.15 (1.18-3.93)	0.024	
Hyperlipidaemia	No	2.63 (1.64-4.2)	<0.001	0.357	1.84 (0.86-3.93)	0.189	0.413	0.84 (0.45-1.59)	0.642	<0.001
	0-6 months	2.86 (1.39-5.91)	0.009		1.15 (0.29-4.49)	0.933		1.46 (0.84-2.55)	0.246	
	>6-36 months	8.58 (1.9-38.79)	0.011		4.13 (1.18-14.48)	0.073		9.45 (3.84-23.25)	<0.001	
	>3 years	4.65 (2.15-10.06)	<0.001		3.05 (1.48-6.26)	0.019		1.86 (1.26-2.74)	0.006	
Respiratory	No	3.82 (2.57-5.69)	<0.001	0.036	4.4 (2.55-7.6)	<0.001	<0.001	1.91 (1.37-2.67)	0.001	0.307
	0-6 months	0.98 (0.39-2.45)	0.997			0.988		0.96 (0.48-1.92)	0.936	
	>6-36 months	7.76 (1.03-58.24)	0.072		0.59 (0.13-2.76)	0.611		1.48 (0.56-3.91)	0.494	
	>3 years	3.93 (0.89-17.24)	0.106		1.51 (0.44-5.21)	0.611		1.78 (1.02-3.09)	0.071	
Renal	No	3.21 (2.22-4.66)	<0.001	0.435	2.82 (1.72-4.62)	<0.001	0.027	1.69 (1.23-2.32)	0.005	0.217
	0-6 months	5.47 (1.21-24.78)	0.048					3.14 (0.67-14.66)	0.207	
	>6-36 months							0.71 (0.26-1.95)	0.574	
	>3 years	2.25 (0.4-12.87)	0.434		2.03 (0.5-8.18)	0.431		2.33 (1.21-4.49)	0.023	

Pancreas (Acute)	No							1.54 (1.15-2.05)	0.011	<0.001
	0-6 months							0.43 (0.03-6.26)	0.595	
	>6-36 months							53.27 (7.44-381.61)	0.001	
	>3 years							243.98 (31.23-1906.12)	<0.001	
Pancreas (Chronic)	No							1.53 (1.14-2.05)	0.012	<0.001
	0-6 months							1.83 (0.48-6.95)	0.456	
	>6-36 months							26.06 (5.12-132.59)	0.001	
	>3 years							12.3 (2.05-73.82)	0.015	
Liver (Chronic)	No				1.95 (1.2-3.18)	0.036	0.1	1.5 (1.11-2.01)	0.017	0.014
	0-6 months				6.61 (0.95-46.05)	0.12		3.44 (1.03-11.56)	0.073	
	>6-36 months				16.76 (2.11-133.45)	0.036		6.73 (1.55-29.21)	0.023	
	>3 years				16.76 (0.71-395.47)	0.141		5.97 (1.98-18.04)	0.005	
Biliary (Chronic)	No	3.04 (2.12-4.36)	<0.001	0.012	2.2 (1.36-3.56)	0.013	0.504	1.6 (1.18-2.16)	0.007	0.247
	0-6 months	3.26 (0.55-19.26)	0.245		4.21 (0.53-33.42)	0.254		1.75 (0.73-4.15)	0.273	
	>6-36 months				11.04 (0.87-139.74)	0.122		5.74 (1.7-19.38)	0.012	
	>3 years							2.1 (0.68-6.44)	0.263	
Lower GI	No	3.4 (2.33-4.96)	<0.001	0.037	2.02 (1.19-3.44)	0.036	0.636	1.65 (1.22-2.24)	0.005	0.049
	0-6 months	1.18 (0.3-4.62)	0.908		2.55 (0.59-10.91)	0.291		0.72 (0.27-1.98)	0.595	
	>6-36 months	35.42 (3.64-345.02)	0.005		5.78 (0.95-35.18)	0.12		5.42 (1.72-17.04)	0.011	
	>3 years	1 (0.17-6.05)	0.997		3.94 (0.93-16.67)	0.122		2.29 (1.05-4.96)	0.062	
Smoker	Never	4.47 (2.38-8.36)	<0.001	0.482	2.55 (1.18-5.52)	0.056	0.848	2.04 (1.37-3.04)	0.002	0.008
	Past	2.39 (1.04-5.48)	0.063		1.73 (0.67-4.41)	0.349		1.44 (0.92-2.27)	0.161	
	Recent	2.06 (0.81-5.23)	0.183		3.03 (1.21-7.59)	0.056		2.41 (1.4-4.13)	0.005	
Obese	Never	3.26 (1.85-5.75)	<0.001	0.834	3.91 (2.01-7.6)	<0.001	0.162	2.06 (1.37-3.1)	0.002	<0.001
	Past	3.38 (0.18-63.47)	0.491		2.86 (0.23-35.33)	0.532		1.91 (0.68-5.37)	0.287	
	Recent	2.37 (1.13-4.97)	0.041		1.7 (0.75-3.85)	0.291		2.1 (1.41-3.15)	0.002	
					**Hypertension (Ref. No)**					
**Modifier**	**Subgroup**	**0-6 months**			**>6-36 months**			**>3 years**		
		**OR (95% CI)**	**P value**	**P-inter**	**OR (95% CI)**	**P value**	**P-inter**	**OR (95% CI)**	**P value**	**P-inter**
Gender	Female	0.61 (0.37-1.02)	0.433	0.241	0.24 (0.1-0.59)	0.044	0.001	0.8 (0.56-1.13)	0.407	0.789

	Male	0.9 (0.58-1.39)	0.904		1.3 (0.66-2.58)	0.775		0.84 (0.6-1.17)	0.536	
Pancreas (Acute)	No							0.73 (0.55-0.97)	0.174	<0.001
	0-6 months							4.28 (0.63-29.11)	0.295	
	>6-36 months							3.25 (0.59-17.89)	0.366	
	>3 years							20.13 (3.02-134.16)	0.021	
Pancreas (Chronic)	No				0.62 (0.34-1.13)	0.602	0.156	0.72 (0.54-0.95)	0.16	<0.001
	0-6 months				0.39 (0.04-3.99)	0.775		2.97 (0.76-11.68)	0.288	
	>6-36 months				0.28 (0.02-5.05)	0.76		34.56 (6.35-188.05)	<0.001	
	>3 years							4.31 (0.71-26)	0.288	
Upper GI	No	0.7 (0.48-1.01)	0.433	0.519	0.53 (0.27-1.04)	0.403	0.287	0.75 (0.55-1.03)	0.261	0.008
	0-6 months	1.22 (0.41-3.63)	0.957		0.48 (0.07-3.18)	0.775		0.33 (0.16-0.7)	0.035	
	>6-36 months	0.72 (0.07-7.09)	0.981		0.47 (0.1-2.28)	0.734		0.93 (0.37-2.38)	0.937	
	>3 years	1.92 (0.42-8.83)	0.764		2.46 (0.61-9.89)	0.636		1.63 (0.87-3.05)	0.29	

		Hyperlipidaemia (Ref. No)								
**Modifier**	**Subgroup**	**0-6 months**			**>6-36 months**			**>3 years**		
		**OR (95% CI)**	**P value**	**P-inter**	**OR (95% CI)**	**P value**	**P-inter**	**OR (95% CI)**	**P value**	**P-inter**
Diabetes	No	1.08 (0.72-1.63)	0.994	0.465	0.32 (0.16-0.65)	0.036	<0.001	1.02 (0.74-1.4)	0.927	0.106
	0-6 months	1.07 (0.5-2.3)	1		1.02 (0.24-4.44)	0.978		1.69 (0.71-4.01)	0.505	
	>6-36 months	0.55 (0.12-2.47)	0.968		0.71 (0.2-2.52)	0.857		1.57 (0.58-4.25)	0.625	
	>3 years	1.81 (0.85-3.86)	0.735		3.73 (1.52-9.17)	0.072		2.32 (1.18-4.56)	0.097	
Pancreas (Acute)	No				0.69 (0.43-1.1)	0.383	<0.001	1.12 (0.85-1.49)	0.642	<0.001
	0-6 months				0.35 (0.04-3.36)	0.699		1.95 (0.31-12.42)	0.664	
	>6-36 months				1.65 (0.1-26.43)	0.951		7.91 (1.42-43.96)	0.097	
	>3 years							34.78 (4.24-285.63)	0.014	
Pancreas (Chronic)	No				0.59 (0.36-0.96)	0.26	<0.001	1.09 (0.82-1.44)	0.696	<0.001
	0-6 months				1.86 (0.25-13.71)	0.817		1.96 (0.51-7.48)	0.556	
	>6-36 months				11.5 (1.08-122.16)	0.26		59.95 (8-449)	0.002	
	>3 years							25.66 (2.93-224.65)	0.036	
Liver (Chronic)	No	1.1 (0.79-1.53)	0.968	0.364	0.62 (0.38-1)	0.26	<0.001	1.12 (0.84-1.49)	0.642	0.038
	0-6 months	2.13 (0.45-10.02)	0.929		0.51 (0.07-3.5)	0.796		1.76 (0.57-5.46)	0.556	

	>6-36 months				6.18 (0.81-47.07)	0.31		3.56 (0.62-20.34)	0.425	
	>3 years	11.8 (0.58-238.34)	0.694		88.03 (6.39-1211.99)	0.029		5.08 (1.54-16.8)	0.073	

		Respiratory (Ref. No)								
Modifier	Subgroup	0-6 months			>6-36 months			>3 years		
		OR (95% CI)	P value	P-inter	OR (95% CI)	P value	P-inter	OR (95% CI)	P value	P-inter
Pancreas (Acute)	No	0.8 (0.59-1.09)	0.686	0.022	0.74 (0.46-1.2)	0.722	0.098	0.48 (0.35-0.65)	<0.001	0.019
	0-6 months	1.67 (0.09-30.74)	0.935		0.08 (0-2.58)	0.653		0.24 (0.03-2.25)	0.293	
	>6-36 months				2.33 (0.17-31.99)	0.871		11.09 (1.47-83.61)	0.041	
	>3 years							1.25 (0.2-7.92)	0.898	
Pancreas (Chronic)	No				0.71 (0.44-1.16)	0.653	0.166	0.51 (0.38-0.69)	<0.001	0.021
	0-6 months				0.99 (0.13-7.41)	0.991		0.33 (0.08-1.46)	0.238	
	>6-36 months				10.95 (0.42-286.46)	0.653		3.61 (0.71-18.37)	0.212	
	>3 years									
Smoker	Never	0.85 (0.49-1.49)	0.914	0.283	1.76 (0.8-3.85)	0.653	0.037	0.86 (0.53-1.4)	0.664	0.04
	Past	1.24 (0.72-2.14)	0.854		0.78 (0.33-1.85)	0.871		0.38 (0.22-0.65)	0.003	
	Recent	0.78 (0.39-1.55)	0.86		0.66 (0.29-1.47)	0.788		0.51 (0.3-0.87)	0.029	
Substance user	Never							0.87 (0.46-1.64)	0.775	0.008
	Recent							0.15 (0.06-0.36)	<0.001	
Obese	Never	1.14 (0.74-1.78)	0.914	0.262	1.7 (0.89-3.22)	0.653	0.014	0.58 (0.39-0.89)	0.028	0.08
	Past	1.02 (0.16-6.47)	0.998		1.1 (0.15-8.37)	0.991		0.76 (0.22-2.61)	0.775	
	Recent	0.57 (0.32-1.03)	0.462		0.37 (0.16-0.85)	0.339		0.55 (0.35-0.88)	0.029	

		Cardiovascular (Ref. No)								
Modifier	Subgroup	0-6 months			>6-36 months			>3 years		
		OR (95% CI)	P value	P-inter	OR (95% CI)	P value	P-inter	OR (95% CI)	P value	P-inter
Obese	Never	0.58 (0.36-0.93)	0.147	0.712	0.4 (0.2-0.83)	0.061	0.554	0.5 (0.31-0.8)	0.017	0.009
	Past	1.25 (0.28-5.52)	0.885		0.47 (0.05-4.26)	0.632		0.61 (0.19-1.96)	0.572	
	Recent	0.77 (0.45-1.32)	0.511		0.63 (0.31-1.3)	0.372		0.82 (0.49-1.35)	0.591	

		Pancreas (Acute) (Ref. No)								
Modifier	Subgroup	0-6 months			>6-36 months			>3 years		

		OR (95% CI)	P value	P-inter	OR (95% CI)	P value	P-inter	OR (95% CI)	P value	P-inter
Ethnicity	White				0.2 (0.05-0.86)	0.245	<0.001	0.55 (0.18-1.73)	0.733	0.317
	South Asian				43.56 (7.02-270.24)	0.001		2.57 (0.57-11.62)	0.679	
	Black				0.42 (0.04-4.1)	0.991		1.29 (0.13-12.39)	0.989	
Diagnosis age group	18-40			0.039	27.06 (3.82-191.81)	0.013	0.003	1.25 (0.08-19.68)	0.989	0.96
	41-50	0.71 (0.02-26.24)	0.993		0.03 (0-0.4)	0.082		0.34 (0.03-3.86)	0.791	
	51-60	0.54 (0.15-2)	0.832		1.28 (0.36-4.53)	0.991		0.87 (0.21-3.55)	0.989	
	61-70	18.64 (1.68-206.43)	0.265		0.64 (0.06-6.66)	0.991		0.79 (0.13-4.66)	0.989	
	71-80	0.91 (0.04-22.1)	0.993		1.19 (0.19-7.56)	0.991		1.26 (0.25-6.22)	0.989	
	>80	9.76 (0.96-99.07)	0.558					1.35 (0.11-16.64)	0.989	
Obese	Never	4.69 (1.56-14.07)	0.12	0.031	0.34 (0.1-1.13)	0.434	0.03	0.36 (0.1-1.23)	0.411	<0.001
	Past				15.95 (0.66-385.01)	0.441				
	Recent	0.32 (0.06-1.79)	0.645		2.03 (0.51-8.16)	0.874		8.06 (1.96-33.18)	0.055	

		Pancreas (Chronic) (Ref. No)								
Modifier	Subgroup	0-6 months			>6-36 months			>3 years		
		OR (95% CI)	P value	P-inter	OR (95% CI)	P value	P-inter	OR (95% CI)	P value	P-inter
Gender	Female	34.03 (15.41-75.14)	<0.001	0.365	8.99 (2.73-29.64)	0.002	0.169	15.02 (3.45-65.36)	0.004	0.071
	Male	59.89 (22.71-157.97)	<0.001		3.24 (1.22-8.63)	0.044		3.1 (1.05-9.12)	0.081	
Ethnicity	White	28.25 (13.52-59.01)	<0.001	0.002	3.42 (1.24-9.4)	0.042	<0.001	4.02 (1.2-13.45)	0.071	0.761
	South Asian				327.45 (22.18-4834.13)	<0.001		6.95 (1.18-40.87)	0.081	
	Black	122.24 (29.48-506.97)	<0.001		2.96 (0.31-28.61)	0.479		5.41 (0.42-69.4)	0.321	
Diagnosis age group	18-40	119.8 (9.8-1464.24)	<0.001	0.359	33.23 (3.03-364.35)	0.017	0.034	4.36 (0.32-58.87)	0.426	0.524
	41-50	11.48 (2.87-46)	<0.001		1.21 (0.1-14.73)	0.987		1.3 (0.13-12.6)	0.99	
	51-60	56.68 (16.19-198.47)	<0.001		5.7 (1.64-19.8)	0.018		13.27 (2.56-68.73)	0.012	
	61-70	88.06 (17.62-439.95)	<0.001		9.12 (1.94-42.92)	0.017		2.84 (0.23-34.9)	0.639	
	71-80	29.05 (5.85-144.24)	<0.001					4.88 (0.75-31.69)	0.182	
	>80	51.03 (10.62-245.1)	<0.001		16.06 (1.93-133.57)	0.027		14.74 (1.2-181)	0.081	
Diabetes	No	32.68 (14.97-71.34)	<0.001	0.368	1.99 (0.72-5.55)	0.324	0.01			
	0-6 months									
	>6-36 months	154.08 (15.93-1490.35)	<0.001		5.71 (0.39-84.56)	0.33				

	>3 years	41.52 (13.06-132)	<0.001		30.12 (7.25-125.16)	<0.001				
Hypertension	No	19.39 (6.8-55.3)	<0.001	0.108				1.71 (0.42-6.95)	0.681	0.038
	0-6 months	40.05 (4.36-367.68)	0.002							
	>6-36 months	13.59 (2.37-77.98)	0.004							
	>3 years	86.2 (34.17-217.46)	<0.001					10.07 (2.76-36.74)	0.004	
Hyperlipidaemia	No	22.13 (8.59-57)	<0.001	0.013	0.47 (0.09-2.54)	0.509	<0.001	0.92 (0.19-4.47)	0.99	<0.001
	0-6 months									
	>6-36 months	60.46 (10.53-347.24)	<0.001		7.18 (1.36-37.74)	0.045				
	>3 years	36.85 (13.87-97.89)	<0.001		20.44 (6.46-64.72)	<0.001		21.67 (4.53-103.73)	0.002	
Pancreas (Acute)	No	52.12 (25.26-107.57)	<0.001	0.021	4.16 (1.52-11.35)	0.017	0.42			
	0-6 months	72.78 (7.06-750.19)	<0.001							
	>6-36 months	1.19 (0.08-17.02)	0.974		2.9 (0.58-14.54)	0.33				
	>3 years	209.78 (7.3-6024.07)	0.003		25.06 (1.54-406.8)	0.051				
Biliary(Chronic)	No	72 (33.57-154.43)	<0.001	<0.001	3.98 (1.54-10.32)	0.017	0.865	4.04 (1.44-11.38)	0.032	0.798
	0-6 months	3.03 (0.91-10.05)	0.081		4.57 (0.31-67.42)	0.377		7.42 (0.62-89.41)	0.208	
	>6-36 months				6.62 (1.15-38.26)	0.073		7.22 (0.59-88.31)	0.214	
	>3 years	38.61 (3.55-420.04)	0.004		11.34 (0.95-135.57)	0.112		13.67 (1.21-154.4)	0.081	
Upper GI	No	44.12 (19.07-102.1)	<0.001	0.84	1.63 (0.5-5.31)	0.535	0.007	3.62 (1.08-12.17)	0.081	0.032
	0-6 months	92.39 (9.55-894.04)	<0.001							
	>6-36 months	53.9 (4.33-671.22)	0.003		3.46 (0.47-25.31)	0.33				
	>3 years	31.68 (10.47-95.9)	<0.001		19.18 (4.72-77.89)	<0.001		4.56 (1.19-17.46)	0.072	
Lower GI	No	32.15 (15.54-66.52)	<0.001	0.004	4.19 (1.71-10.29)	0.009	0.274			
	0-6 months									
	>6-36 months	10.25 (1.88-56.06)	0.009		9 (0.42-193.68)	0.296				
	>3 years				2.8 (0.27-29)	0.509				
Smoker	Never	34.42 (12.33-96.07)	<0.001	0.855	7.89 (2.55-24.4)	0.002	0.003	4.31 (1.22-15.18)	0.071	0.384
	Past	53.28 (18.42-154.12)	<0.001		11.83 (1.62-86.34)	0.038		4.31 (0.86-21.64)	0.15	
	Recent	53.13 (15.53-181.83)	<0.001		9.22 (2.23-38.07)	0.011		19.56 (3.16-121.03)	0.009	
Drinker	Never	25.98 (8.32-81.17)	<0.001	0.206	111.44 (14.02-885.76)	<0.001	<0.001	4.76 (1.08-20.96)	0.081	0.678
	Recent	70.98 (25.67-196.21)	<0.001		6.42 (1.76-23.37)	0.017		6.49 (1.61-26.27)	0.032	

Obese	Never	36.11 (15.58-83.68)	<0.001	0.684	1.89 (0.68-5.25)	0.33	0.006	4.31 (1.5-12.42)	0.032	0.036
	Past	20.23 (1.95-209.35)	0.014					35.2 (2.49-498.35)	0.032	
	Recent	55.97 (21.15-148.14)	<0.001		24.49 (6.25-96)	<0.001		33.2 (3.95-278.77)	0.009	

		Liver (Chronic) (Ref. No)								
Modifier	Subgroup	0-6 months			>6-36 months			>3 years		
		OR (95% CI)	P value	P-inter	OR (95% CI)	P value	P-inter	OR (95% CI)	P value	P-inter
Gender	Female	3.48 (1.58-7.7)	0.009	0.734	0.26 (0.09-0.72)	0.04	0.743	0.5 (0.25-1.02)	0.129	0.015
	Male	2.92 (1.55-5.52)	0.006		0.33 (0.13-0.81)	0.057		0.14 (0.06-0.3)	<0.001	
Ethnicity	White	1.6 (0.82-3.1)	0.248	0.012	0.27 (0.12-0.65)	0.019	0.681	0.21 (0.1-0.41)	<0.001	0.025
	South Asian	4.23 (0.92-19.53)	0.114		0.81 (0.17-3.79)	0.992		0.9 (0.35-2.29)	0.991	
	Black	10.54 (2.44-45.63)	0.009		0.2 (0.02-2.1)	0.365		0.1 (0.01-0.91)	0.093	
Hyperlipidaemia	No	2.52 (1.19-5.36)	0.041	0.793	0.11 (0.03-0.47)	0.019	0.151	0.1 (0.04-0.25)	<0.001	0.003
	0-6 months	4.61 (1.25-16.97)	0.046					0.88 (0.06-13.69)	0.991	
	>6-36 months	2.18 (0.39-12.23)	0.484		1.01 (0.22-4.65)	0.992		4.26 (0.51-35.55)	0.356	
	>3 years	3.82 (1.61-9.06)	0.009		0.37 (0.14-0.99)	0.142		0.43 (0.2-0.93)	0.076	
Respiratory	No	3.75 (2.02-6.93)	<0.001	0.404	0.32 (0.14-0.7)	0.024	0.934	0.16 (0.08-0.32)	<0.001	0.002
	0-6 months	5.65 (0.92-34.76)	0.113		1.43 (0-9260.13)	0.992		1.06 (0.06-17.38)	0.991	
	>6-36 months	4.12 (0.23-75.17)	0.446		0.16 (0.01-1.85)	0.321				
	>3 years	1.39 (0.44-4.34)	0.654		0.27 (0.05-1.37)	0.273		0.34 (0.13-0.94)	0.09	
Cardiovascular	No	3.09 (1.71-5.58)	0.001	0.764	0.23 (0.1-0.51)	0.007	0.261	0.21 (0.12-0.4)	<0.001	<0.001
	0-6 months	2.3 (0.61-8.7)	0.312		5.09 (0.34-75.3)	0.453				
	>6-36 months	1.95 (0.2-18.86)	0.654		0.57 (0.07-4.72)	0.897		3.79 (0.44-32.48)	0.409	
	>3 years	6.18 (1.35-28.23)	0.045		0.44 (0.05-3.5)	0.712		0.07 (0.01-0.51)	0.027	
Pancreas (Acute)	No	3 (1.79-5.04)	<0.001	0.171	0.28 (0.14-0.6)	0.008	0.625	0.22 (0.13-0.39)	<0.001	0.035
	0-6 months	1.13 (0.09-13.75)	0.987							
	>6-36 months				0.77 (0.08-7.6)	0.992				
	>3 years							3.01 (0.48-19.06)	0.429	
Upper GI	No	2.21 (1.13-4.33)	0.046	0.184	0.26 (0.1-0.63)	0.019	0.572	0.09 (0.04-0.25)	<0.001	<0.001
	0-6 months	8.97 (2.06-39.18)	0.012		1.66 (0.13-21.65)	0.992		2.66 (0.59-12.02)	0.391	
	>6-36 months	8.03 (1.81-35.63)	0.018		0.4 (0.07-2.42)	0.595		1.51 (0.11-21.33)	0.991	

	>3 years	2.51 (0.81-7.73)	0.181		0.21 (0.04-1.14)	0.184		0.29 (0.11-0.76)	0.031	
Lower GI	No	2.48 (1.39-4.41)	0.009	0.318	0.24 (0.11-0.55)	0.008	0.09	0.23 (0.13-0.42)	<0.001	<0.001
	0-6 months	14.97 (1.5-149.52)	0.046							
	>6-36 months	6.11 (1.19-31.38)	0.063					2.48 (0.19-32.08)	0.785	
	>3 years	3.8 (0.82-17.67)	0.153		0.46 (0.08-2.74)	0.675		0.05 (0-0.51)	0.031	
Obese	Never	3.11 (1.41-6.84)	0.016	0.952	0.14 (0.05-0.44)	0.008	0.006	0.11 (0.05-0.27)	<0.001	<0.001
	Past	7.77 (0.34-176.57)	0.287		1.17 (0.09-15.73)	0.992		1.78 (0.18-17.72)	0.941	
	Recent	3.11 (1.39-6.94)	0.018		1.06 (0.41-2.75)	0.992		1.04 (0.48-2.24)	0.991	

		Biliary (Chronic) (Ref. No)								
**Modifier**	**Subgroup**	**0-6 months**			**>6-36 months**			**>3 years**		
		**OR (95% CI)**	**P value**	**P-inter**	**OR (95% CI)**	**P value**	**P-inter**	**OR (95% CI)**	**P value**	**P-inter**
Hyperlipidaemia	No	9.33 (5.01-17.38)	<0.001	0.011	0.44 (0.16-1.2)	0.392	0.092	0.28 (0.12-0.65)	0.028	0.084
	0-6 months	32.07 (9.82-104.79)	<0.001		10.21 (0.79-131.82)	0.392		5.14 (0.31-84.39)	0.514	
	>6-36 months	14.95 (4.86-45.98)	<0.001		1.25 (0.27-5.78)	0.989		2.06 (0.04-95.33)	0.988	
	>3 years	4.12 (2.09-8.12)	<0.001		0.48 (0.18-1.23)	0.392		0.77 (0.36-1.66)	0.864	
Respiratory	No	9.14 (5.62-14.85)	<0.001	0.868	0.59 (0.29-1.19)	0.392	0.538	0.32 (0.16-0.67)	0.027	0.037
	0-6 months	7.77 (2.5-24.08)	<0.001					6.52 (0.41-102.91)	0.469	
	>6-36 months	15.05 (3.4-66.56)	<0.001		0.18 (0.01-2.36)	0.421				
	>3 years	7.32 (2.77-19.34)	<0.001		1.12 (0.36-3.46)	0.989		0.82 (0.33-2.04)	0.988	
Pancreas (Chronic)	No	10.56 (6.97-16.01)	<0.001	0.001	0.56 (0.29-1.1)	0.392	0.775	0.48 (0.27-0.85)	0.07	0.657
	0-6 months	0.48 (0.13-1.82)	0.29					0.25 (0.02-2.81)	0.518	
	>6-36 months	12.05 (0.69-209.51)	0.099		0.79 (0.13-4.84)	0.989		1.1 (0.08-14.76)	0.988	
	>3 years	20.51 (1.49-281.82)	0.029		1.12 (0.09-14.16)	0.989		1.73 (0.15-19.93)	0.988	
Lower GI	No	9.06 (5.77-14.21)	<0.001	0.309	0.57 (0.3-1.08)	0.392	0.114	0.36 (0.19-0.68)	0.027	<0.001
	0-6 months	40.18 (4.48-360.58)	0.001		1.44 (0.01-239.1)	0.989		145 (7.19-2922.41)	0.023	
	>6-36 months	3.35 (0.46-24.34)	0.245		4.69 (0.81-27.06)	0.392				
	>3 years	7.04 (2.32-21.44)	<0.001					0.8 (0.25-2.58)	0.988	
Obese	Never	18.46 (9.49-35.95)	<0.001	0.012	0.56 (0.23-1.36)	0.421	0.177	0.51 (0.24-1.08)	0.251	0.046
	Past	21.61 (4.22-110.63)	<0.001					5.68 (0.63-51.38)	0.343	
	Recent	5.17 (2.74-9.76)	<0.001		1.16 (0.49-2.74)	0.989		0.69 (0.28-1.73)	0.77	

Modifier	Subgroup	Upper GI (Ref. No) 0-6 months OR (95% CI)	P value	P-inter	>6-36 months OR (95% CI)	P value	P-inter	>3 years OR (95% CI)	P value	P-inter
Pancreas (Acute)	No	4.12 (2.92-5.83)	<0.001	<0.001				0.87 (0.66-1.15)	0.885	0.058
	0-6 months	15.79 (0.76-327.56)	0.091					1.33 (0.2-8.76)	0.945	
	>6-36 months							5.94 (0.87-40.36)	0.486	
	>3 years							4.59 (0.88-24.03)	0.486	
Pancreas (Chronic)	No	4.09 (2.88-5.79)	<0.001	<0.001	1.21 (0.76-1.91)	0.912	0.748	0.9 (0.68-1.18)	0.892	0.024
	0-6 months	9.56 (0.74-122.78)	0.1		1.6 (0.13-19.61)	0.917		0.75 (0.19-2.95)	0.911	
	>6-36 months				2.25 (0.22-23.2)	0.912		13.35 (2.25-79.05)	0.107	
	>3 years							0.97 (0.18-5.26)	0.999	
Liver (Chronic)	No	3.57 (2.5-5.11)	<0.001	<0.001	1.07 (0.67-1.73)	0.955	0.131	0.9 (0.68-1.19)	0.9	0.446
	0-6 months	12.48 (2.53-61.48)	0.003		4.84 (0.97-24.12)	0.531		1.02 (0.28-3.73)	0.999	
	>6-36 months	14.34 (0.9-227.41)	0.073		1.95 (0.3-12.87)	0.912		0.68 (0.1-4.71)	0.911	
	>3 years	129.49 (21.4-783.63)	<0.001		14.14 (0.79-251.83)	0.531		2.74 (0.72-10.45)	0.63	
Biliary (Chronic)	No	4.23 (2.97-6.03)	<0.001	0.005	1.19 (0.74-1.93)	0.912	0.172	0.94 (0.71-1.25)	0.911	0.35
	0-6 months	1.74 (0.44-6.87)	0.463		1.58 (0.41-6.09)	0.912		0.47 (0.17-1.33)	0.646	
	>6-36 months	137.36 (10.69-1764.95)	<0.001		0.61 (0.06-6.2)	0.912		1.5 (0.38-5.95)	0.911	
	>3 years	30.03 (2.9-311.23)	0.007					1.73 (0.57-5.31)	0.885	
Lower GI	No	4.44 (3.02-6.53)	<0.001	0.006	1.29 (0.79-2.13)	0.835	0.287	0.78 (0.58-1.06)	0.63	0.067
	0-6 months	1.52 (0.57-4.04)	0.435		4.68 (0.94-23.31)	0.531		1.43 (0.56-3.65)	0.9	
	>6-36 months	52.12 (7.1-382.7)	<0.001		0.64 (0.14-2.95)	0.912		2.5 (0.77-8.15)	0.63	
	>3 years	8.32 (2.74-25.27)	<0.001		0.69 (0.12-3.91)	0.912		1.69 (0.78-3.63)	0.646	

Modifier	Subgroup	Lower GI (Ref. No) 0-6 months OR (95% CI)	P value	P-inter	>6-36 months OR (95% CI)	P value	P-inter	>3 years OR (95% CI)	P value	P-inter
Biliary (Chronic)	No	0.68 (0.44-1.06)	0.458	0.001	0.49 (0.29-0.84)	0.086	0.065	0.53 (0.35-0.79)	0.017	0.38
	0-6 months	3.17 (0.35-28.85)	0.771		0.21 (0.03-1.45)	0.326		0.47 (0.15-1.49)	0.385	
	>6-36 months	2.46 (0.02-285.38)	0.92		3.6 (0.59-22.04)	0.403				
	>3 years	219.58 (10.09-4778.65)	0.013					1.16 (0.33-4.13)	0.992	

Smoker	Never	0.91 (0.45-1.8)	0.92	0.089	0.82 (0.39-1.73)	0.884	0.461	0.41 (0.21-0.8)	0.046	0.038
	Past	1.38 (0.66-2.92)	0.79		0.46 (0.19-1.1)	0.261		0.79 (0.44-1.41)	0.693	
	Recent	0.84 (0.36-1.95)	0.914		0.48 (0.13-1.73)	0.463		0.6 (0.3-1.22)	0.34	

Modifier	Subgroup	**Smoker (Ref. Never)** Past			Recent		
		**OR (95% CI)**	**P value**	**P-inter**	**OR (95% CI)**	**P value**	**P-inter**
Pancreas (Acute)	No	1.25 (0.94-1.67)	0.357	0.043	1.77 (1.29-2.42)	0.003	0.408
	0-6 months	0.63 (0.1-4.04)	0.828		1.56 (0.18-13.29)	0.782	
	>6-36 months	5.14 (0.37-71.69)	0.466		1.01 (0.14-7.33)	0.999	
	>3 years	23.88 (2.31-246.97)	0.202		10.57 (1.11-101)	0.119	

Modifier	Subgroup	**Drinker (Ref. Never)** Past			Recent		
		**OR (95% CI)**	**P value**	**P-inter**	**OR (95% CI)**	**P value**	**P-inter**
Ethnicity	White	1.01 (0.44-2.36)	1	0.929	2.42 (1.56-3.76)	0.002	0.022
	South Asian	1.14 (0.14-9.26)	1		1.38 (0.63-3.01)	0.564	
	Black	1.6 (0.41-6.27)	1		1.83 (0.87-3.85)	0.224	
Diagnosis age group	18-40	2.94 (0.53-16.17)	1	0.891	0.38 (0.1-1.44)	0.256	0.043
	41-50	1.6 (0.25-10.06)	1		0.92 (0.38-2.26)	0.888	
	51-60	1.05 (0.23-4.79)	1		2.89 (1.51-5.55)	0.008	
	61-70	0.76 (0.21-2.69)	1		1.5 (0.87-2.57)	0.251	
	71-80	1.4 (0.43-4.59)	1		2.21 (1.19-4.1)	0.046	
	>80	1.12 (0.24-5.21)	1		2.25 (1.05-4.83)	0.092	
Pancreas (Acute)	No				1.73 (1.26-2.37)	0.007	0.026
	0-6 months				24.83 (1.91-322.26)	0.047	
	>6-36 months				0.12 (0.01-1.92)	0.246	
	>3 years				3.49 (0.5-24.2)	0.327	
Pancreas (Chronic)	No	1.12 (0.58-2.16)	1	0.472	1.8 (1.31-2.48)	0.005	0.035
	0-6 months				4.25 (0.95-18.96)	0.128	
	>6-36 months				0.05 (0-0.81)	0.091	
	>3 years				2.28 (0.3-17.2)	0.564	

Modifier	Subgroup	Substance user (Ref. Never)					
		Past			Recent		
		OR (95% CI)	P value	P-inter	OR (95% CI)	P value	P-inter
Respiratory	No				2.91 (1.73-4.88)	0.004	0.002
	0-6 months				1.66 (0.63-4.37)	0.517	
	>6-36 months				1.59 (0.39-6.4)	0.678	
	>3 years				0.32 (0.1-0.96)	0.134	
Renal	No				2.09 (1.33-3.29)	0.02	0.032
	0-6 months				0.2 (0.02-2.17)	0.388	
	>6-36 months				13.58 (0.96-191.37)	0.152	
	>3 years				0.69 (0.19-2.57)	0.721	

Modifier	Subgroup	Obese (Ref. Never)					
		Past			Recent		
		OR (95% CI)	P value	P-inter	OR (95% CI)	P value	P-inter
Ethnicity	White				0.4 (0.3-0.55)	<0.001	0.046
	South Asian				0.7 (0.39-1.28)	0.307	
	Black				0.74 (0.42-1.3)	0.344	
Respiratory	No	0.61 (0.31-1.2)	0.611	0.908	0.52 (0.39-0.69)	<0.001	0.015
	0-6 months	0.45 (0.07-3.1)	0.71		0.26 (0.13-0.51)	<0.001	
	>6-36 months	0.46 (0.06-3.85)	0.764		0.14 (0.05-0.36)	<0.001	
	>3 years	0.88 (0.29-2.74)	0.994		0.53 (0.31-0.93)	0.04	
Pancreas (Acute)	No	0.5 (0.28-0.9)	0.48	0.003	0.43 (0.34-0.55)	<0.001	<0.001
	0-6 months				0.05 (0.01-0.35)	0.005	
	>6-36 months	19.92 (0.37-1064.75)	0.609		2.24 (0.39-12.91)	0.426	
	>3 years				7.37 (1.24-43.86)	0.043	
Pancreas (Chronic)	No	0.57 (0.32-1.02)	0.583	0.156	0.41 (0.32-0.53)	<0.001	0.006
	0-6 months	0.43 (0.04-5.12)	0.782		0.62 (0.18-2.1)	0.489	
	>6-36 months				5.18 (1.07-25.11)	0.061	
	>3 years	6.05 (0.33-111.98)	0.693		2.54 (0.27-23.63)	0.47	
Liver (Chronic)	No	0.52 (0.29-0.94)	0.48	0.115	0.4 (0.31-0.51)	<0.001	<0.001

	0-6 months	1.45 (0.06-34.18)	0.994		0.43 (0.15-1.28)	0.168	
	>6-36 months	3.6 (0.17-75.01)	0.71		3.29 (0.76-14.25)	0.15	
	>3 years	9.46 (0.74-120.46)	0.583		2.9 (0.92-9.13)	0.096	
Biliary (Chronic)	No	0.58 (0.32-1.06)	0.583	0.167	0.47 (0.36-0.6)	<0.001	0.038
	0-6 months	0.92 (0.16-5.23)	0.994		0.14 (0.06-0.35)	<0.001	
	>6-36 months				0.89 (0.27-2.93)	0.853	
	>3 years	6.02 (0.6-60.01)	0.609		0.63 (0.2-2)	0.489	

OR adjusted for all demographics, comorbidities and lifestyle factors. The reported P values are corrected for multiple testing via Benjamini-Hochberg method.

Interaction between groups evaluated by the likelihood ratio test. Only those predictor-modifier combinations are shown if at least one interaction among predictor-modifier interaction pairs shows significance (P value for interaction<0.05). Empty cells represent situations when less than 5 patients present to calculate the interaction.

PC, pancreatic cancer; GI, gastrointestinal tract; OR, odds ratio; CI, confidence interval.

Table 3 Association between study variables and odds of pancreatic cancer in comparison to non-malignant pancreatic disease group.

	PC (N=965)	PnC (N=3963)	Model$_{PRD_V+AGE_V}$		Model$_{ALL_V}$	
**Demographic**			**OR (95% CI)**	**P value**	**OR (95% CI)**	**P value**
**Gender**						
Female	453 (46.9%)	1847 (46.6%)	Ref.		Ref.	
Male	512 (53.1%)	2116 (53.4%)	1.04 (0.89-1.21)	0.607	0.96 (0.81-1.14)	0.814
**Ethnicity**						
White	537 (55.6%)	2103 (53.1%)	Ref.		Ref.	
South Asian	86 (8.9%)	793 (20.0%)	0.47 (0.36-0.6)	<0.001	0.67 (0.5-0.89)	0.02
Black	100 (10.4%)	366 (9.2%)	1.27 (0.98-1.65)	0.081	1.43 (1.08-1.89)	0.033
Other	85 (8.8%)	421 (10.6%)	0.89 (0.68-1.16)	0.445	0.95 (0.72-1.26)	0.857
Not known	157 (16.3%)	280 (7.1%)	1.75 (1.38-2.2)	<0.001	1.11 (0.85-1.46)	0.548
**Diagnosis age group**						
18-40	26 (2.7%)	1158 (29.2%)	Ref.		Ref.	
41-50	70 (7.3%)	771 (19.5%)	3.71 (2.37-5.98)	<0.001	4.5 (2.82-7.19)	<0.001
51-60	198 (20.5%)	698 (17.6%)	11.6 (7.73-18)	<0.001	15.2 (9.82-23.5)	<0.001
61-70	299 (31.0%)	528 (13.3%)	23.2 (15.6-36)	<0.001	35.3 (22.7-54.8)	<0.001
71-80	246 (25.5%)	470 (11.9%)	21.3 (14.3-33.2)	<0.001	38.2 (24.3-60.1)	<0.001
>80	126 (13.1%)	338 (8.5%)	15.5 (10.2-24.6)	<0.001	33.1 (20.3-53.9)	<0.001
**Comorbidities**						
**Diabetes**						
No	605 (62.7%)	2715 (68.5%)	Ref.		Ref.	
0-6 months	140 (14.5%)	289 (7.3%)	1.74 (1.36-2.22)	<0.001	1.73 (1.32-2.25)	<0.001
>6-36 months	44 (4.6%)	202 (5.1%)	0.89 (0.62-1.27)	0.583	1.33 (0.9-1.96)	0.221
>3 years	176 (18.2%)	757 (19.1%)	0.73 (0.59-0.91)	0.006	1.21 (0.94-1.55)	0.208
**Hypertension**						
No	396 (41.0%)	1687 (42.6%)	Ref.		Ref.	
0-6 months	150 (15.5%)	412 (10.4%)	0.84 (0.66-1.07)	0.193	0.86 (0.66-1.11)	0.352
>6-36 months	32 (3.3%)	301 (7.6%)	0.41 (0.27-0.62)	<0.001	0.51 (0.33-0.79)	0.01
>3 years	387 (40.1%)	1563 (39.4%)	0.51 (0.42-0.62)	<0.001	0.72 (0.56-0.93)	0.033
**Hyperlipidaemia**						
No	524 (54.3%)	2233 (56.3%)	Ref.		Ref.	
0-6 months	148 (15.3%)	519 (13.1%)	0.71 (0.56-0.89)	0.004	0.83 (0.64-1.07)	0.221
>6-36 months	50 (5.2%)	280 (7.1%)	0.58 (0.42-0.82)	0.003	0.82 (0.56-1.19)	0.382
>3 years	243 (25.2%)	931 (23.5%)	0.65 (0.54-0.79)	<0.001	1.02 (0.8-1.3)	0.974
**Respiratory**						
No	719 (74.5%)	2691 (67.9%)	Ref.		Ref.	
0-6 months	106 (11.0%)	420 (10.6%)	0.73 (0.57-0.93)	0.017	0.81 (0.62-1.05)	0.181
>6-36 months	42 (4.4%)	217 (5.5%)	0.66 (0.46-0.94)	0.028	0.84 (0.58-1.24)	0.495
>3 years	98 (10.2%)	635 (16.0%)	0.49 (0.38-0.63)	<0.001	0.62 (0.48-0.82)	0.002
**Renal**						

No	848 (87.9%)	3404 (85.9%)	Ref.		Ref.	
0-6 months	18 (1.9%)	89 (2.2%)	0.47 (0.28-0.81)	0.007	0.44 (0.25-0.77)	0.013
>6-36 months	26 (2.7%)	132 (3.3%)	0.5 (0.32-0.78)	0.003	0.68 (0.42-1.09)	0.181
>3 years	73 (7.6%)	338 (8.5%)	0.51 (0.38-0.68)	<0.001	0.71 (0.52-0.98)	0.072
**Cardiovascular**						
No	714 (74.0%)	2973 (75.0%)	Ref.		Ref.	
0-6 months	127 (13.2%)	361 (9.1%)	0.71 (0.56-0.89)	0.005	0.78 (0.6-1)	0.104
>6-36 months	37 (3.8%)	179 (4.5%)	0.48 (0.33-0.71)	<0.001	0.67 (0.44-1.02)	0.114
>3 years	87 (9.0%)	450 (11.4%)	0.39 (0.3-0.51)	<0.001	0.51 (0.38-0.69)	<0.001
**Liver (Chronic)**						
No	862 (89.3%)	3077 (77.6%)	Ref.		Ref.	
0-6 months	70 (7.3%)	473 (11.9%)	0.54 (0.41-0.72)	<0.001	0.52 (0.39-0.7)	<0.001
>6-36 months	14 (1.5%)	178 (4.5%)	0.33 (0.18-0.57)	<0.001	0.42 (0.23-0.76)	0.013
>3 years	19 (2.0%)	235 (5.9%)	0.3 (0.18-0.49)	<0.001	0.36 (0.22-0.6)	<0.001
**Biliary (Chronic)**						
No	793 (82.2%)	2988 (75.4%)	Ref.		Ref.	
0-6 months	134 (13.9%)	649 (16.4%)	0.68 (0.55-0.84)	<0.001	0.67 (0.53-0.84)	0.002
>6-36 months	18 (1.9%)	163 (4.1%)	0.38 (0.23-0.63)	<0.001	0.54 (0.32-0.92)	0.051
>3 years	20 (2.1%)	163 (4.1%)	0.41 (0.25-0.66)	<0.001	0.54 (0.33-0.9)	0.043
**Upper GI**						
No	644 (66.7%)	2525 (63.7%)	Ref.		Ref.	
0-6 months	144 (14.9%)	261 (6.6%)	2.18 (1.7-2.8)	<0.001	2.61 (2-3.41)	<0.001
>6-36 months	43 (4.5%)	319 (8.0%)	0.56 (0.4-0.8)	0.002	0.75 (0.52-1.08)	0.196
>3 years	134 (13.9%)	858 (21.7%)	0.56 (0.45-0.69)	<0.001	0.72 (0.57-0.92)	0.025
**Lower GI**						
No	815 (84.5%)	3322 (83.8%)	Ref.		Ref.	
0-6 months	66 (6.8%)	165 (4.2%)	1.39 (1.01-1.91)	0.061	1.53 (1.08-2.17)	0.037
>6-36 months	29 (3.0%)	162 (4.1%)	0.68 (0.44-1.04)	0.085	0.93 (0.6-1.46)	0.87
>3 years	55 (5.7%)	314 (7.9%)	0.66 (0.48-0.9)	0.013	0.89 (0.63-1.25)	0.616
**Lifestyle**						
**Smoker**						
Never	257 (26.6%)	1317 (33.2%)	Ref.		Ref.	
Past	194 (20.1%)	636 (16.0%)	1.06 (0.85-1.33)	0.702	1.27 (0.99-1.63)	0.107
Recent	188 (19.5%)	1090 (27.5%)	1.04 (0.82-1.3)	0.766	1.17 (0.91-1.5)	0.328
Not known	326 (33.8%)	920 (23.2%)	1.38 (1.12-1.72)	0.005	0.98 (0.74-1.29)	0.974
**Drinker**						
Never	135 (14.0%)	843 (21.3%)	Ref.		Ref.	
Past	27 (2.8%)	112 (2.8%)	1.17 (0.72-1.91)	0.606	1.34 (0.79-2.25)	0.377
Recent	289 (29.9%)	1432 (36.1%)	1.18 (0.92-1.51)	0.234	1.24 (0.95-1.62)	0.181
Not known	514 (53.3%)	1576 (39.8%)	1.57 (1.25-1.97)	<0.001	1.43 (1.1-1.86)	0.021
**Substance user**						
Never	132 (13.7%)	692 (17.5%)	Ref.		Ref.	
Past	0 (0.0%)	4 (0.1%)	NA	NA	NA	NA

Recent	130 (13.5%)	578 (14.6%)	0.85 (0.63-1.13)	0.33	1 (0.73-1.38)	0.992
Not known	703 (72.8%)	2689 (67.9%)	1.14 (0.92-1.43)	0.33	1 (0.78-1.28)	0.992
**Obese**						
Never	452 (46.8%)	2008 (50.7%)	Ref.		Ref.	
Past	30 (3.1%)	136 (3.4%)	0.75 (0.49-1.15)	0.232	0.97 (0.62-1.53)	0.974
Recent	214 (22.2%)	999 (25.2%)	0.79 (0.65-0.96)	0.027	1 (0.81-1.23)	0.992
Not known	269 (27.9%)	820 (20.7%)	0.99 (0.82-1.2)	0.939	0.76 (0.6-0.96)	0.044

The reported P values are corrected for multiple testing via Benjamini-Hochberg method.
$Model_{PRD\_V+AGE\_V}$: Adjusted for gender, ethnicity, and diagnosis age group variables (AGE_V) for each individual predictor variable (PRD_V)
$Model_{ALL\_V}$: Simultaneously adjusted for all demographic, comorbidities and lifestyle factors
PC, pancreatic cancer; PnC, non-malignant pancreatic disease; GI, gastrointestinal tract; OR, odds ratio; CI, confidence interval; NA, not available.

Table 4 Modified odds of pancreatic cancer associated with individual medical condition and lifestyle factors among different participant subgroups, in comparison to non-malignant pancreatic disease group.

		Diabetes (Ref. No)								
		0-6 months			>6-36 months			>3 years		
**Modifier**	**Subgroup**	**OR (95% CI)**	**P value**	**P-inter**	**OR (95% CI)**	**P value**	**P-inter**	**OR (95% CI)**	**P value**	**P-inter**
Diagnosis age group	18-40			0.091			0.003	3.81 (1.24-11.76)	0.196	0.215
	41-50	1.09 (0.37-3.18)	0.951		2.58 (0.92-7.22)	0.295		1.82 (0.81-4.08)	0.381	
	51-60	2.25 (1.33-3.82)	0.012		1.21 (0.57-2.59)	0.838		1.25 (0.78-2.02)	0.599	
	61-70	1.78 (1.11-2.86)	0.051		1.44 (0.65-3.17)	0.696		0.99 (0.65-1.51)	0.999	
	71-80	1.83 (1.04-3.22)	0.077		0.37 (0.1-1.35)	0.368		1.02 (0.66-1.58)	0.999	
	>80	1.91 (0.87-4.19)	0.175		5.41 (2.24-13.05)	0.011		1.63 (0.96-2.75)	0.261	
Hyperlipidaemia	No	1.52 (1.08-2.14)	0.051	0.031	0.89 (0.45-1.75)	0.881	0.034	0.62 (0.34-1.12)	0.338	<0.001
	0-6 months	1.31 (0.74-2.31)	0.475		0.85 (0.26-2.75)	0.937		0.92 (0.57-1.5)	0.89	
	>6-36 months	9.7 (2.07-45.39)	0.015		4.39 (1.59-12.15)	0.092		5.61 (2.53-12.46)	0.001	
	>3 years	3.23 (1.58-6.6)	0.008		2.14 (1.13-4.06)	0.187		1.42 (1-2.03)	0.238	
Obese	Never	1.92 (1.26-2.92)	0.012	0.639	1.7 (1.01-2.86)	0.233	0.845	1.08 (0.77-1.53)	0.848	<0.001
	Past	5.21 (0.18-149.93)	0.47		1.72 (0.12-24.07)	0.881		1.69 (0.69-4.17)	0.471	
	Recent	2.06 (1.08-3.93)	0.062		1.15 (0.55-2.39)	0.881		1.8 (1.23-2.63)	0.052	

		Hypertension (Ref. No)								
**Modifier**	**Subgroup**	0-6 months			>6-36 months			>3 years		
		**OR (95% CI)**	**P value**	**P-inter**	**OR (95% CI)**	**P value**	**P-inter**	**OR (95% CI)**	**P value**	**P-inter**
Diagnosis age group	18-40	0.97 (0.22-4.29)	0.982	0.421			0.533	1.51 (0.5-4.55)	0.68	0.037
	41-50	0.53 (0.2-1.41)	0.982		0.89 (0.32-2.47)	0.984		0.51 (0.26-0.99)	0.132	
	51-60	0.9 (0.52-1.54)	0.982		0.57 (0.25-1.31)	0.412		0.64 (0.43-0.95)	0.086	
	61-70	0.85 (0.52-1.38)	0.982		0.45 (0.15-1.32)	0.391		0.6 (0.4-0.9)	0.05	
	71-80	0.9 (0.52-1.54)	0.982		0.63 (0.25-1.59)	0.63		0.73 (0.46-1.17)	0.376	
	>80	2.1 (0.87-5.02)	0.982		0.28 (0.05-1.54)	0.391		1.89 (0.9-3.96)	0.203	

Obese	Never	0.65 (0.43-1)	0.815	0.021	0.35 (0.18-0.68)	0.057	0.029	0.65 (0.48-0.88)	0.029	<0.001
	Past							0.77 (0.18-3.29)	0.861	
	Recent	1.35 (0.64-2.86)	0.982		1.7 (0.72-4.04)	0.467		1.35 (0.82-2.24)	0.449	
					**Hyperlipidaemia (Ref. No)**					
**Modifier**	**Subgroup**	**0-6 months**			**>6-36 months**			**>3 years**		
		**OR (95% CI)**	**P value**	**P-inter**	**OR (95% CI)**	**P value**	**P-inter**	**OR (95% CI)**	**P value**	**P-inter**
Diabetes	No	0.81 (0.57-1.16)	0.824	0.622	0.3 (0.16-0.56)	0.011	<0.001	0.79 (0.57-1.08)	0.683	0.014
	0-6 months	0.75 (0.43-1.32)	0.847		1.8 (0.42-7.7)	0.704		1.54 (0.71-3.34)	0.765	
	>6-36 months	0.91 (0.24-3.46)	0.943		1.63 (0.56-4.76)	0.704		2.01 (0.82-4.89)	0.683	
	>3 years	1.3 (0.65-2.58)	0.93		2.7 (1.2-6.07)	0.333		1.81 (0.96-3.41)	0.683	
					**Cardiovascular (Ref. No)**					
**Modifier**	**Subgroup**	**0-6 months**			**>6-36 months**			**>3 years**		
		**OR (95% CI)**		**P-inter**	**OR (95% CI)**		**P-inter**	**OR (95% CI)**		**P-inter**
Obese	Never	0.75 (0.52-1.1)	0.442	0.576	0.65 (0.35-1.19)	0.648	0.538	0.47 (0.31-0.71)	0.004	0.023
	Past	0.97 (0.26-3.71)	0.999		0.57 (0.1-3.28)	0.95		1.02 (0.37-2.78)	0.995	
	Recent	1 (0.62-1.63)	0.999		0.96 (0.49-1.89)	0.993		0.7 (0.43-1.12)	0.207	
					**Liver (Chronic) (Ref. No)**					
**Modifier**	**Subgroup**	**0-6 months**			**>6-36 months**			**>3 years**		
		**OR (95% CI)**	**P value**	**P-inter**	**OR (95% CI)**	**P value**	**P-inter**	**OR (95% CI)**	**P value**	**P-inter**
Obese	Never	0.49 (0.32-0.75)	0.007	0.609	0.36 (0.14-0.92)	0.24	0.326	0.19 (0.08-0.45)	0.004	0.016
	Past	0.52 (0.06-4.52)	0.69		0.98 (0.07-13.77)	0.998		0.35 (0.07-1.78)	0.403	
	Recent	0.68 (0.41-1.14)	0.259		0.62 (0.27-1.41)	0.541		0.9 (0.44-1.83)	0.963	
					**Biliary (Chronic) (Ref. No)**					
**Modifier**	**Subgroup**	**0-6 months**			**>6-36 months**			**>3 years**		
		**OR (95% CI)**	**P value**	**P-inter**	**OR (95% CI)**	**P value**	**P-inter**	**OR (95% CI)**	**P value**	**P-inter**
Gender	Female	0.52 (0.37-0.71)	0.001	0.022	0.41 (0.2-0.84)	0.164	0.316	0.45 (0.24-0.86)	0.168	0.338
	Male	0.88 (0.63-1.21)	0.602		0.71 (0.32-1.6)	0.695		0.77 (0.32-1.84)	0.929	

Hyperlipidaemia	No	0.55 (0.39-0.77)	0.004	0.012	0.35 (0.13-0.94)	0.27	0.46	0.9 (0.37-2.18)	0.982	0.466
	0-6 months	1.23 (0.72-2.08)	0.629		0.83 (0.25-2.72)	0.929		0.25 (0.06-1.1)	0.274	
	>6-36 months	1.34 (0.62-2.91)	0.629		1.18 (0.29-4.74)	0.975		0.45 (0.05-4.17)	0.852	
	>3 years	0.5 (0.32-0.78)	0.011		0.43 (0.17-1.07)	0.338		0.51 (0.24-1.07)	0.284	
Upper GI	No	0.78 (0.59-1.02)	0.154	0.009	0.4 (0.16-0.96)	0.278	0.126	0.44 (0.21-0.94)	0.176	0.629
	0-6 months	0.28 (0.15-0.52)	0.001		1.84 (0.55-6.2)	0.621		0.31 (0.06-1.68)	0.483	
	>6-36 months	1.28 (0.54-3.03)	0.741		0.17 (0.02-1.41)	0.338		1.13 (0.21-6.15)	0.982	
	>3 years	0.56 (0.3-1.03)	0.14		0.48 (0.18-1.3)	0.375		0.71 (0.3-1.66)	0.852	
Smoker	Never	0.78 (0.53-1.15)	0.39	<0.001	0.24 (0.08-0.7)	0.164	0.014	0.79 (0.39-1.61)	0.886	0.383
	Past	0.44 (0.27-0.72)	0.007		0.45 (0.17-1.24)	0.338		0.28 (0.1-0.75)	0.168	
	Recent	1.43 (0.88-2.31)	0.299		2.06 (0.82-5.19)	0.338		0.59 (0.16-2.15)	0.852	

		Upper GI (Ref. No)								
Modifier	Subgroup	0-6 months			>6-36 months			>3 years		
		**OR (95% CI)**	**P value**	**P-inter**	**OR (95% CI)**	**P value**	**P-inter**	**OR (95% CI)**	**P value**	**P-inter**
Gender	Female	1.98 (1.33-2.93)	0.002	0.029	1.05 (0.64-1.73)	0.956	0.081	0.87 (0.62-1.2)	0.648	0.141
	Male	3.54 (2.45-5.11)	<0.001		0.55 (0.3-0.98)	0.733		0.62 (0.44-0.87)	0.129	
Ethnicity	White	2.76 (1.94-3.92)	<0.001	0.174	0.7 (0.42-1.18)	0.733	0.277	0.73 (0.54-0.99)	0.176	0.888
	South Asian	3.36 (1.62-6.93)	0.003		0.86 (0.37-2.01)	0.956		0.72 (0.4-1.28)	0.487	
	Black	1.13 (0.49-2.57)	0.78		1.03 (0.39-2.72)	0.979		0.66 (0.34-1.28)	0.45	
Hyperlipidaemia	No	3.33 (2.23-4.97)	<0.001	0.019	0.6 (0.31-1.17)	0.733	0.045	0.53 (0.34-0.85)	0.129	0.009
	0-6 months	1.26 (0.69-2.27)	0.482		0.24 (0.07-0.84)	0.733		0.48 (0.26-0.87)	0.13	
	>6-36 months	2.01 (0.78-5.19)	0.184		1.13 (0.39-3.24)	0.956		0.4 (0.15-1.03)	0.184	
	>3 years	3.88 (2.26-6.65)	<0.001		1.26 (0.71-2.23)	0.881		1.14 (0.8-1.63)	0.712	
Biliary (Chronic)	No	3.21 (2.34-4.42)	<0.001	0.003	0.71 (0.45-1.1)	0.733	0.49	0.75 (0.57-0.99)	0.176	0.689
	0-6 months	1.15 (0.62-2.11)	0.67		1.17 (0.51-2.67)	0.956		0.55 (0.3-1.04)	0.196	
	>6-36 months	13.21 (3.04-57.5)	0.002		0.34 (0.04-3.11)	0.789		0.87 (0.24-3.23)	0.951	
	>3 years	2.05 (0.35-12.12)	0.474		1.71 (0.28-10.51)	0.881		1.11 (0.37-3.35)	0.951	
Smoker	Never	1.56 (0.95-2.55)	0.107	0.02	0.65 (0.34-1.26)	0.733	0.915	0.97 (0.66-1.42)	0.951	0.328

	Past	2.43 (1.38-4.27)	0.005		0.85 (0.43-1.68)	0.908		0.61 (0.4-0.95)	0.162	
	Recent	4.5 (2.69-7.51)	<0.001		0.9 (0.46-1.77)	0.956		0.66 (0.41-1.06)	0.249	
Drinker	Never	2.46 (1.31-4.6)	0.009	0.01	1.15 (0.47-2.83)	0.956	0.769	0.96 (0.58-1.59)	0.951	0.139
	Past	8.05 (1.82-35.63)	0.01		1.19 (0.19-7.63)	0.957		0.34 (0.09-1.39)	0.309	
	Recent	4.13 (2.7-6.31)	<0.001		0.76 (0.42-1.36)	0.79		0.88 (0.61-1.28)	0.748	

Modifier	Subgroup	Smoker (Ref. Never)					
		Past			Recent		
		OR (95% CI)	P value	P-inter	OR (95% CI)	P value	P-inter
Biliary (Chronic)	No	1.35 (1-1.83)	0.584	0.116	1.02 (0.76-1.38)	0.905	0.002
	0-6 months	0.79 (0.44-1.43)	0.805		1.84 (1.03-3.28)	0.505	
	>6-36 months	2.53 (0.6-10.74)	0.636		9.99 (2.52-39.6)	0.023	
	>3 years	0.52 (0.16-1.69)	0.701		0.59 (0.14-2.54)	0.844	
Upper GI	No	1.21 (0.86-1.7)	0.701	0.132	1.08 (0.78-1.51)	0.851	0.002
	0-6 months	1.88 (0.93-3.79)	0.622		3.32 (1.73-6.38)	0.02	
	>6-36 months	2.04 (0.84-5)	0.622		1.54 (0.63-3.77)	0.742	
	>3 years	0.81 (0.49-1.33)	0.805		0.71 (0.42-1.21)	0.621	

OR adjusted for all demographics, comorbidities and lifestyle factors. The reported P values are corrected for multiple testing via Benjamini-Hochberg method.

Interaction between groups evaluated by the likelihood ratio test. Only those predictor-modifier combinations are shown if at least one interaction among predictor-modifier interaction pairs shows significance (P value for interaction<0.05). Empty cells represent situations when less than 5 patients present to calculate the interaction.

GI, gastrointestinal tract; OR, odds ratio; CI, confidence interval.
