# Supplementary material for: Temporality of clinical factors associated with pancreatic cancer: a case-control study using linked electronic health records
Source: BMC Cancer. 2021 Nov 27;21:1279. doi: 10.1186/s12885-021-09014-w (PMC8626898; doi:10.1186/s12885-021-09014-w)
Supplement: Supplementary file 3 — Additional file 3: Sensitivity Analyses Results. Fig 1. Sensitivity analysis: Forest plots showing association between study variables and odds of pancreatic cancer in comparison to control group. Fig 2. Sensitivity analysis: Forest plots showing association between study variables and odds of pancreatic cancer in comparison to non-malignant pancreatic disease group. Table 1. Sensitivity analysis: Association between study variables and odds of pancreatic cancer in comparison to chronic pancreatic conditions group. [file 12885_2021_9014_MOESM3_ESM.pdf]

Contents

Fig. 1 Sensitivity analysis: Forest plots showing association between study variables and odds of pancreatic cancer in comparison to control group. 2

Fig. 2 Sensitivity analysis: Forest plots showing association between study variables and odds of pancreatic cancer in comparison to non-malignant pancreatic disease group. 4

Table 1 Sensitivity analysis: Association between study variables and odds of pancreatic cancer in comparison to chronic pancreatic conditions group. 6

A.

	OR	95% CI	P val
**Demography**			
**Gender (ref=Female)**			
Male	0.74	0.59-0.94	0.029
**Ethnicity (ref=White)**			
South Asian	1.06	0.75-1.51	0.828
Black	1.04	0.75-1.43	0.867
Other	1.08	0.73-1.61	0.811
**Diagnosis age group (ref=18-40)**			
41-50	2.65	1.43-4.89	0.005
51-60	6.2	3.5-11	<0.001
61-70	11	6.14-19.6	<0.001
71-80	16.1	8.79-29.6	<0.001
>80	14.6	7.7-27.8	<0.001
**Medical History (ref=No)**			
**Diabetes**			
0-6 months	3.76	2.33-6.06	<0.001
>6-36 months	2.01	1.2-3.37	0.018
>3 years	1.8	1.34-2.4	<0.001
**Hypertension**			
0-6 months	1.01	0.57-1.79	0.970
>6-36 months	0.86	0.47-1.59	0.788
>3 years	1.12	0.82-1.53	0.578
**Hyperlipidaemia**			
0-6 months	1.23	0.85-1.77	0.373
>6-36 months	0.9	0.56-1.44	0.792
>3 years	1.38	1.04-1.83	0.052
**Respiratory**			
0-6 months	0.86	0.59-1.25	0.559
>6-36 months	0.95	0.59-1.52	0.867
>3 years	0.57	0.42-0.77	<0.001
**Renal**			
0-6 months	0.24	0.1-0.57	0.003
>6-36 months	0.6	0.34-1.04	0.126
>3 years	0.76	0.54-1.09	0.221
**Cardiovascular**			
0-6 months	0.63	0.43-0.91	0.029
>6-36 months	0.49	0.29-0.83	0.017
>3 years	0.5	0.35-0.7	<0.001
**Pancreas (Acute)**			
0-6 months	2.07	0.87-4.92	0.167
>6-36 months	0.87	0.34-2.24	0.860
>3 years	1.53	0.63-3.68	0.454
**Pancreas (Chronic)**			
0-6 months	31.6	16.6-60	<0.001
>6-36 months	6.77	2.94-15.6	<0.001
>3 years	4.64	1.79-12	0.004
**Liver (Chronic)**			
0-6 months	3.06	1.75-5.37	<0.001
>6-36 months	0.34	0.16-0.71	0.010
>3 years	0.4	0.23-0.7	0.004
**Biliary (Chronic)**			
0-6 months	8.28	5.33-12.8	<0.001
>6-36 months	0.66	0.34-1.24	0.296
>3 years	0.68	0.39-1.19	0.281
**Upper GI**			
0-6 months	4.08	2.84-5.86	<0.001
>6-36 months	1.32	0.84-2.07	0.319
>3 years	0.95	0.72-1.25	0.811
**Lower GI**			
0-6 months	1.06	0.67-1.66	0.867
>6-36 months	0.59	0.35-1	0.092
>3 years	0.55	0.38-0.8	0.005
**Lifestyle (ref=Never)**			
**Smoker**			
Past	1.29	0.97-1.72	0.134
Recent	1.71	1.25-2.33	0.002
**Drinker**			
Past	1.35	0.76-2.42	0.420
Recent	2.01	1.46-2.76	<0.001
**Substance user**			
Recent	1.55	1.03-2.35	0.070
**Obese**			
Past	0.67	0.4-1.13	0.220
Recent	0.49	0.38-0.64	<0.001

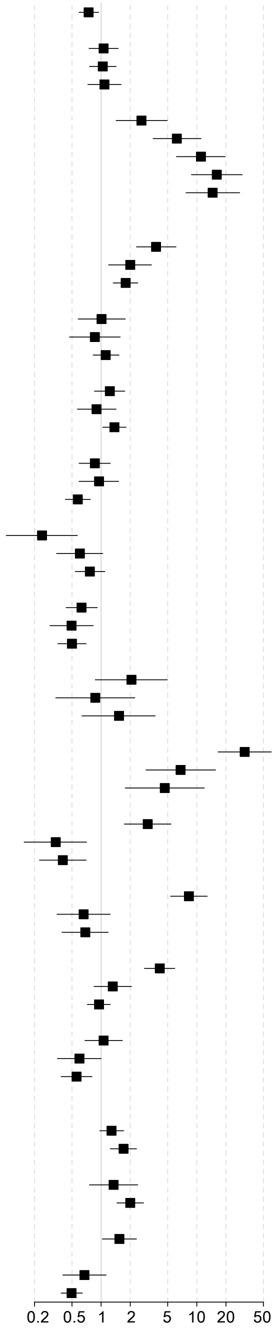

B.

	OR	95% CI	P val
**Demography**			
**Gender (ref=Female)**			
Male	0.89	0.74-1.07	0.292
**Ethnicity (ref=White)**			
South Asian	0.81	0.6-1.1	0.233
Black	0.85	0.64-1.13	0.324
Other	1.12	0.82-1.52	0.541
**Diagnosis age group (ref=18-40)**			
41-50	3.31	2.02-5.43	<0.001
51-60	8.78	5.56-13.9	<0.001
61-70	20.1	12.7-31.7	<0.001
71-80	31.1	19.3-50.2	<0.001
>80	21.3	12.8-35.5	<0.001
**Medical History (ref=No)**			
**Diabetes**			
0-6 months	3.22	2.33-4.46	<0.001
>6-36 months	2.04	1.33-3.12	0.002
>3 years	1.61	1.24-2.1	0.001
**Hypertension**			
0-6 months	0.74	0.55-1.01	0.096
>6-36 months	0.41	0.25-0.66	<0.001
>3 years	0.64	0.5-0.83	0.001
**Hyperlipidaemia**			
0-6 months	0.94	0.7-1.25	0.711
>6-36 months	0.65	0.43-0.98	0.065
>3 years	1.06	0.83-1.36	0.696
**Respiratory**			
0-6 months	0.81	0.61-1.08	0.214
>6-36 months	0.8	0.52-1.24	0.364
>3 years	0.51	0.38-0.68	<0.001
**Renal**			
0-6 months	0.22	0.12-0.42	<0.001
>6-36 months	0.68	0.4-1.13	0.196
>3 years	0.84	0.6-1.18	0.364
**Cardiovascular**			
0-6 months	0.65	0.48-0.86	0.006
>6-36 months	0.51	0.32-0.81	0.007
>3 years	0.57	0.42-0.79	0.002
**Pancreas (Acute)**			
0-6 months	1.5	0.68-3.31	0.364
>6-36 months	0.94	0.4-2.21	0.906
>3 years	0.98	0.44-2.2	0.964
**Pancreas (Chronic)**			
0-6 months	40	21.7-73.5	<0.001
>6-36 months	4.89	2.25-10.6	<0.001
>3 years	5.18	2.23-12	<0.001
**Liver (Chronic)**			
0-6 months	2.94	1.8-4.81	<0.001
>6-36 months	0.32	0.16-0.62	0.002
>3 years	0.3	0.18-0.52	<0.001
**Biliary (Chronic)**			
0-6 months	7.76	5.29-11.4	<0.001
>6-36 months	0.58	0.32-1.03	0.096
>3 years	0.44	0.26-0.74	0.004
**Upper GI**			
0-6 months	3.99	2.9-5.48	<0.001
>6-36 months	1.05	0.7-1.59	0.837
>3 years	0.86	0.67-1.11	0.315
**Lower GI**			
0-6 months	0.81	0.54-1.21	0.362
>6-36 months	0.53	0.32-0.88	0.023
>3 years	0.52	0.36-0.74	<0.001
**Lifestyle (ref=Non-recent)**			
**Smoker**			
Recent	1.32	1.04-1.67	0.035
**Drinker**			
Recent	1.2	0.98-1.47	0.124
**Substance user**			
Recent	0.78	0.59-1.02	0.106
**Obese**			
Recent	0.51	0.41-0.63	<0.001

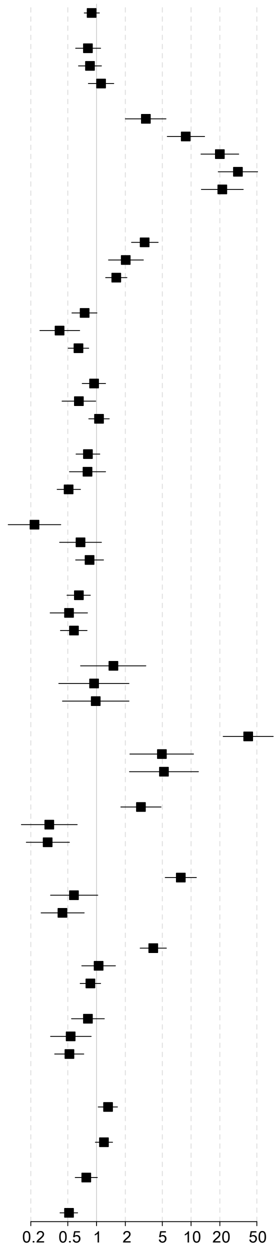

Fig. 1 Sensitivity analysis: Forest plots showing association between study variables and odds of pancreatic cancer (PC) in comparison to control group. (A) Sensitivity Analysis 1 conducted with patients having both primary and secondary care records (PC, *N*=566; control, N=3793). (B) Sensitivity Analysis 2 conducted with dichotomised lifestyle data imputed with *past*, *never* and *missing* categories are collapsed into *non-recent* category (PC, *N*=965; control, N=4355).

A.

	OR	95% CI	P val
**Demography**			
**Gender (ref=Female)**			
Male	0.91	0.73-1.13	0.525
**Ethnicity (ref=White)**			
South Asian	0.68	0.5-0.94	0.048
Black	1.58	1.16-2.15	0.015
Other	1.01	0.71-1.44	0.959
**Diagnosis age group (ref=18-40)**			
41-50	4.03	2.27-7.15	<0.001
51-60	12.8	7.48-21.8	<0.001
61-70	30	17.3-52	<0.001
71-80	35	19.7-62.2	<0.001
>80	34	18.5-62.2	<0.001
**Medical History (ref=No)**			
**Diabetes**			
0-6 months	2.4	1.57-3.68	<0.001
>6-36 months	1.17	0.75-1.82	0.646
>3 years	1.3	1.01-1.69	0.100
**Hypertension**			
0-6 months	0.75	0.46-1.24	0.392
>6-36 months	0.65	0.39-1.11	0.214
>3 years	0.85	0.63-1.14	0.392
**Hyperlipidaemia**			
0-6 months	0.81	0.58-1.13	0.364
>6-36 months	0.9	0.6-1.35	0.732
>3 years	1.07	0.82-1.4	0.732
**Respiratory**			
0-6 months	0.75	0.54-1.06	0.196
>6-36 months	0.97	0.65-1.46	0.935
>3 years	0.67	0.51-0.88	0.016
**Renal**			
0-6 months	0.6	0.28-1.3	0.332
>6-36 months	0.62	0.38-1.03	0.138
>3 years	0.67	0.48-0.92	0.040
**Cardiovascular**			
0-6 months	0.84	0.6-1.16	0.413
>6-36 months	0.66	0.41-1.04	0.144
>3 years	0.51	0.37-0.7	<0.001
**Liver (Chronic)**			
0-6 months	0.51	0.36-0.73	0.001
>6-36 months	0.41	0.22-0.77	0.017
>3 years	0.38	0.22-0.63	<0.001
**Biliary (Chronic)**			
0-6 months	0.69	0.52-0.92	0.033
>6-36 months	0.64	0.36-1.13	0.230
>3 years	0.55	0.33-0.92	0.056
**Upper GI**			
0-6 months	2.66	1.95-3.63	<0.001
>6-36 months	0.74	0.5-1.11	0.256
>3 years	0.76	0.59-0.98	0.077
**Lower GI**			
0-6 months	1.84	1.26-2.7	0.007
>6-36 months	0.96	0.6-1.54	0.922
>3 years	0.97	0.68-1.37	0.922
**Lifestyle (ref=Never)**			
**Smoker**			
Past	1.15	0.88-1.5	0.436
Recent	1.09	0.83-1.44	0.699
**Drinker**			
Past	1.37	0.8-2.36	0.392
Recent	1.41	1.05-1.89	0.056
**Substance user**			
Recent	1.07	0.73-1.56	0.841
**Obese**			
Past	0.89	0.56-1.41	0.732
Recent	0.97	0.77-1.22	0.902

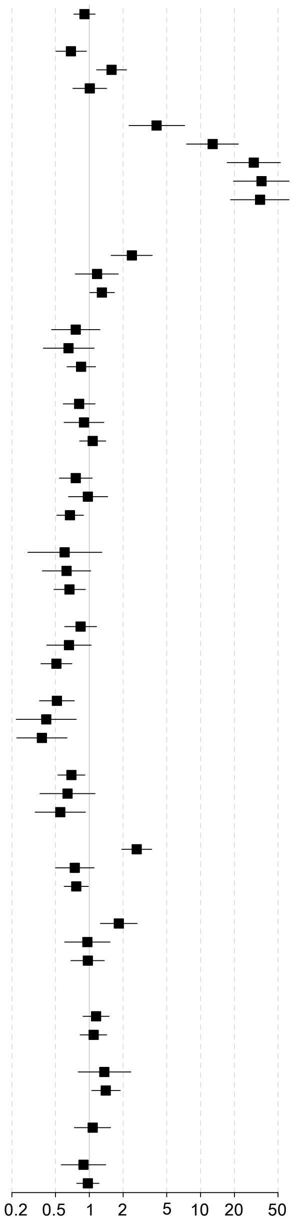

B.

	OR	95% CI	P val
**Demography**			
**Gender (ref=Female)**			
Male	1.01	0.85-1.19	0.940
**Ethnicity (ref=White)**			
South Asian	0.64	0.48-0.85	0.006
Black	1.41	1.06-1.86	0.042
Other	0.92	0.7-1.23	0.688
**Diagnosis age group (ref=18-40)**			
41-50	4.45	2.79-7.1	<0.001
51-60	14.9	9.65-23	<0.001
61-70	34.3	22.1-53	<0.001
71-80	36.9	23.5-57.8	<0.001
>80	32.7	20.1-53.1	<0.001
**Medical History (ref=No)**			
**Diabetes**			
0-6 months	1.68	1.29-2.18	<0.001
>6-36 months	1.3	0.88-1.91	0.267
>3 years	1.18	0.92-1.5	0.268
**Hypertension**			
0-6 months	0.85	0.65-1.1	0.286
>6-36 months	0.5	0.33-0.78	0.006
>3 years	0.76	0.59-0.97	0.055
**Hyperlipidaemia**			
0-6 months	0.84	0.65-1.08	0.267
>6-36 months	0.84	0.58-1.21	0.468
>3 years	1.06	0.83-1.34	0.709
**Respiratory**			
0-6 months	0.8	0.62-1.04	0.163
>6-36 months	0.87	0.59-1.27	0.578
>3 years	0.63	0.49-0.82	0.002
**Renal**			
0-6 months	0.44	0.25-0.77	0.011
>6-36 months	0.68	0.42-1.09	0.174
>3 years	0.73	0.53-0.99	0.086
**Cardiovascular**			
0-6 months	0.78	0.61-1	0.098
>6-36 months	0.69	0.45-1.04	0.129
>3 years	0.51	0.38-0.69	<0.001
**Liver (Chronic)**			
0-6 months	0.51	0.38-0.68	<0.001
>6-36 months	0.42	0.23-0.76	0.011
>3 years	0.36	0.22-0.6	<0.001
**Biliary (Chronic)**			
0-6 months	0.67	0.53-0.84	0.002
>6-36 months	0.54	0.32-0.91	0.044
>3 years	0.55	0.33-0.92	0.044
**Upper GI**			
0-6 months	2.66	2.04-3.47	<0.001
>6-36 months	0.78	0.54-1.13	0.267
>3 years	0.75	0.59-0.95	0.042
**Lower GI**			
0-6 months	1.51	1.07-2.14	0.042
>6-36 months	0.95	0.61-1.49	0.870
>3 years	0.92	0.66-1.29	0.709
**Lifestyle (ref=Non-recent)**			
**Smoker**			
Recent	1.1	0.89-1.36	0.489
**Drinker**			
Recent	1.02	0.84-1.22	0.888
**Substance user**			
Recent	1.05	0.83-1.34	0.709
**Obese**			
Recent	1.06	0.86-1.29	0.688

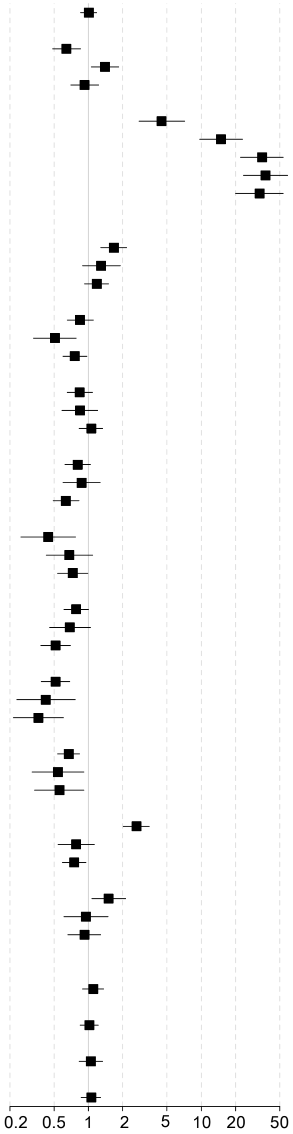

Fig. 2 Sensitivity analysis: Forest plots showing association between study variables and odds of pancreatic cancer (PC) in comparison to non-malignant pancreatic disease group (PnC). (A) Sensitivity Analysis 1 conducted with patients having both primary and secondary care records (PC, *N*=566; PnC, *N*=3038). (B) Sensitivity Analysis 2 conducted with dichotomised lifestyle data imputed with *past*, *never* and *missing* categories are collapsed into *non-recent* category (PC, N=965; PnC, N=3963).

Table 1 Sensitivity analysis: Association between study variables and odds of pancreatic cancer in comparison to chronic pancreatic conditions group.

Demographics	PC (N=965)	PnC (Chronic) (N=2339)	$Model_{PRD\_V+AGE\_V}$ OR (95% CI)	P value	$Model_{ALL\_V}$ OR (95% CI)	P value
**Gender**						
Female	453 (46.9%)	1045 (44.7%)	Ref.		Ref.	
Male	512 (53.1%)	1294 (55.3%)	0.94 (0.8-1.1)	0.479	0.96 (0.8-1.16)	0.786
**Ethnicity**						
White	537 (55.6%)	1328 (56.8%)	Ref.		Ref.	
South Asian	86 (8.9%)	327 (14.0%)	0.71 (0.54-0.93)	0.02	1.06 (0.76-1.46)	0.786
Black	100 (10.4%)	213 (9.1%)	1.3 (0.98-1.71)	0.081	1.42 (1.04-1.93)	0.058
Other	85 (8.8%)	237 (10.1%)	0.99 (0.74-1.31)	0.928	1.06 (0.77-1.45)	0.786
Unknown	157 (16.3%)	234 (10.0%)	1.37 (1.07-1.74)	0.017	0.87 (0.66-1.15)	0.429
**Diagnosis age group**						
18-40	26 (2.7%)	580 (24.8%)	Ref.		Ref.	
41-50	70 (7.3%)	449 (19.2%)	3.34 (2.12-5.43)	<0.001	3.81 (2.35-6.18)	<0.001
51-60	198 (20.5%)	405 (17.3%)	10.5 (6.97-16.5)	<0.001	13.2 (8.36-20.7)	<0.001
61-70	299 (31.0%)	343 (14.7%)	18.8 (12.5-29.3)	<0.001	24.4 (15.5-38.5)	<0.001
71-80	246 (25.5%)	324 (13.9%)	16.1 (10.7-25.3)	<0.001	23 (14.4-36.7)	<0.001
>80	126 (13.1%)	238 (10.2%)	11.5 (7.43-18.3)	<0.001	20 (12-33.3)	<0.001
**Comorbidities**						
**Diabetes**						
No	605 (62.7%)	1509 (64.5%)	Ref.		Ref.	
0-6 months	140 (14.5%)	205 (8.8%)	1.55 (1.19-2)	0.002	1.42 (1.07-1.88)	0.037
>6-36 months	44 (4.6%)	150 (6.4%)	0.79 (0.54-1.15)	0.252	1.15 (0.76-1.75)	0.612
>3 years	176 (18.2%)	475 (20.3%)	0.7 (0.56-0.87)	0.003	0.96 (0.73-1.26)	0.786
**Hypertension**						
No	396 (41.0%)	971 (41.5%)	Ref.		Ref.	
0-6 months	150 (15.5%)	246 (10.5%)	0.91 (0.71-1.18)	0.564	0.95 (0.72-1.27)	0.786
>6-36 months	32 (3.3%)	188 (8.0%)	0.41 (0.27-0.62)	<0.001	0.55 (0.34-0.88)	0.03
>3 years	387 (40.1%)	934 (39.9%)	0.54 (0.44-0.67)	<0.001	0.8 (0.6-1.07)	0.217
**Hyperlipidaemia**						
No	524 (54.3%)	1305 (55.8%)	Ref.		Ref.	
0-6 months	148 (15.3%)	300 (12.8%)	0.77 (0.6-0.98)	0.057	0.92 (0.69-1.22)	0.63
>6-36 months	50 (5.2%)	181 (7.7%)	0.56 (0.39-0.79)	0.002	0.93 (0.62-1.4)	0.786
>3 years	243 (25.2%)	553 (23.6%)	0.71 (0.58-0.88)	0.003	1.2 (0.91-1.57)	0.329
**Respiratory**						
No	719 (74.5%)	1552 (66.4%)	Ref.		Ref.	
0-6 months	106 (11.0%)	267 (11.4%)	0.67 (0.52-0.86)	0.004	0.73 (0.55-0.96)	0.053
>6-36 months	42 (4.4%)	133 (5.7%)	0.64 (0.44-0.94)	0.035	0.81 (0.53-1.23)	0.429
>3 years	98 (10.2%)	387 (16.5%)	0.46 (0.36-0.59)	<0.001	0.6 (0.45-0.8)	0.002
**Renal**						
No	848 (87.9%)	1989 (85.0%)	Ref.		Ref.	
0-6 months	18 (1.9%)	66 (2.8%)	0.44 (0.25-0.75)	0.005	0.38 (0.21-0.69)	0.005
>6-36 months	26 (2.7%)	79 (3.4%)	0.54 (0.34-0.86)	0.014	0.73 (0.44-1.22)	0.361
>3 years	73 (7.6%)	205 (8.8%)	0.53 (0.39-0.71)	<0.001	0.68 (0.48-0.96)	0.058
**Cardiovascular**						
No	714 (74.0%)	1698 (72.6%)	Ref.		Ref.	
0-6 months	127 (13.2%)	260 (11.1%)	0.64 (0.5-0.82)	<0.001	0.66 (0.5-0.86)	0.009
>6-36 months	37 (3.8%)	104 (4.4%)	0.53 (0.35-0.8)	0.004	0.77 (0.48-1.22)	0.37
>3 years	87 (9.0%)	277 (11.8%)	0.4 (0.3-0.52)	<0.001	0.48 (0.35-0.67)	<0.001

**Pancreas (Acute)**						
No	913 (94.6%)	1840 (78.7%)	Ref.		Ref.	
0-6 months	27 (2.8%)	208 (8.9%)	0.35 (0.23-0.54)	<0.001	0.31 (0.2-0.5)	<0.001
>6-36 months	12 (1.2%)	196 (8.4%)	0.18 (0.1-0.33)	<0.001	0.24 (0.13-0.44)	<0.001
>3 years	13 (1.3%)	95 (4.1%)	0.38 (0.21-0.7)	0.003	0.52 (0.27-1)	0.088
**Liver (Chronic)**						
No	862 (89.3%)	1694 (72.4%)	Ref.		Ref.	
0-6 months	70 (7.3%)	328 (14.0%)	0.45 (0.34-0.6)	<0.001	0.43 (0.32-0.59)	<0.001
>6-36 months	14 (1.5%)	140 (6.0%)	0.24 (0.14-0.43)	<0.001	0.35 (0.19-0.65)	0.004
>3 years	19 (2.0%)	177 (7.6%)	0.22 (0.13-0.36)	<0.001	0.27 (0.16-0.46)	<0.001
**Biliary (Chronic)**						
No	793 (82.2%)	1780 (76.1%)	Ref.		Ref.	
0-6 months	134 (13.9%)	353 (15.1%)	0.71 (0.56-0.89)	0.005	0.7 (0.54-0.89)	0.014
>6-36 months	18 (1.9%)	106 (4.5%)	0.33 (0.2-0.56)	<0.001	0.56 (0.31-0.98)	0.08
>3 years	20 (2.1%)	100 (4.3%)	0.35 (0.21-0.58)	<0.001	0.48 (0.28-0.83)	0.024
**Upper GI**						
No	644 (66.7%)	1456 (62.2%)	Ref.		Ref.	
0-6 months	144 (14.9%)	161 (6.9%)	1.93 (1.48-2.52)	<0.001	2.34 (1.73-3.14)	<0.001
>6-36 months	43 (4.5%)	222 (9.5%)	0.44 (0.31-0.63)	<0.001	0.58 (0.39-0.87)	0.024
>3 years	134 (13.9%)	500 (21.4%)	0.52 (0.41-0.65)	<0.001	0.68 (0.52-0.88)	0.012
**Lower GI**						
No	815 (84.5%)	1932 (82.6%)	Ref.		Ref.	
0-6 months	66 (6.8%)	102 (4.4%)	1.31 (0.93-1.85)	0.142	1.51 (1.03-2.21)	0.066
>6-36 months	29 (3.0%)	110 (4.7%)	0.55 (0.36-0.85)	0.013	0.79 (0.49-1.28)	0.44
>3 years	55 (5.7%)	195 (8.3%)	0.6 (0.43-0.84)	0.005	0.8 (0.56-1.16)	0.361
**Lifestyle**						
**Smoker**						
Never	257 (26.6%)	648 (27.7%)	Ref.		Ref.	
Past	194 (20.1%)	355 (15.2%)	1.02 (0.8-1.3)	0.883	1.25 (0.95-1.66)	0.198
Recent	188 (19.5%)	651 (27.8%)	0.81 (0.64-1.04)	0.136	0.98 (0.75-1.3)	0.932
Not known	326 (33.8%)	685 (29.3%)	1.05 (0.84-1.32)	0.763	0.66 (0.49-0.9)	0.025
**Drinker**						
Never	135 (14.0%)	414 (17.7%)	Ref.		Ref.	
Past	27 (2.8%)	61 (2.6%)	1.11 (0.66-1.88)	0.801	1.22 (0.68-2.18)	0.612
Recent	289 (29.9%)	866 (37.0%)	1.02 (0.78-1.32)	0.963	1.19 (0.89-1.6)	0.361
Not known	514 (53.3%)	998 (42.7%)	1.39 (1.09-1.78)	0.016	1.45 (1.09-1.95)	0.03
**Substance user**						
Never	132 (13.7%)	316 (13.5%)	Ref.		Ref.	
Past	0 (0.0%)	2 (0.1%)	NA	NA	NA	NA
Recent	130 (13.5%)	392 (16.8%)	0.67 (0.5-0.92)	0.022	0.86 (0.6-1.22)	0.487
Not known	703 (72.8%)	1629 (69.6%)	0.93 (0.73-1.18)	0.68	0.85 (0.64-1.12)	0.361
**Obese**						
Never	452 (46.8%)	1238 (52.9%)	Ref.		Ref.	
Past	30 (3.1%)	76 (3.2%)	0.8 (0.51-1.26)	0.473	1.26 (0.76-2.11)	0.473
Recent	214 (22.2%)	503 (21.5%)	0.98 (0.8-1.2)	0.902	1.34 (1.05-1.7)	0.038
Not known	269 (27.9%)	522 (22.3%)	1.04 (0.85-1.28)	0.822	0.86 (0.67-1.12)	0.37

The reported P values are corrected for multiple testing via Benjamini-Hochberg method.
$Model_{PRD\_V+AGE\_V}$: Adjusted for gender, ethnicity, and diagnosis age group variables (AGE_V) for each individual predictor variable (PRD_V)
$Model_{ALL\_V}$: Simultaneously adjusted for all demographic, comorbidities and lifestyle factors
PC, pancreatic cancer; PnC, non-malignant pancreatic disease; GI, gastrointestinal tract; OR, odds ratio; CI, confidence interval; NA, not available
